# Trends in incidence of self-harm, neurodevelopmental and mental health conditions among university students compared with the general population: nationwide electronic data linkage study in Wales

**DOI:** 10.1192/bjp.2024.90

**Published:** 2024-09

**Authors:** Ann John, Olivier Y. Rouquette, Sze Chim Lee, Jo Smith, Marcos del Pozo Baños

**Affiliations:** Population Psychiatry Suicide and Informatics, Population Data Science, Swansea University Medical School, Swansea University, Swansea, UK; School of Allied Health and Community, University of Worcester, Worcester, UK

**Keywords:** University students, self-harm, mental health, electronic health records, neurodevelopmental disorders

## Abstract

**Background:**

Concern that self-harm and mental health conditions are increasing in university students may reflect widening access to higher education, existing population trends and/or stressors associated with this setting.

**Aims:**

To compare population-level data on self-harm, neurodevelopmental and mental health conditions between university students and non-students with similar characteristics before and during enrolment.

**Method:**

This cohort study linked electronic records from the Higher Education Statistics Agency for 2012–2018 to primary and secondary healthcare records. Students were undergraduates aged 18 to 24 years at university entry. Non-students were pseudo-randomly selected based on an equivalent age distribution. Logistic regressions were used to calculate odds ratios. Poisson regressions were used to calculate incidence rate ratios (IRR).

**Results:**

The study included 96 760 students and 151 795 non-students. Being male, self-harm and mental health conditions recorded before university entry, and higher deprivation levels, resulted in lower odds of becoming a student and higher odds of drop-out from university. IRRs for self-harm, depression, anxiety, autism spectrum disorder (ASD), drug use and schizophrenia were lower for students. IRRs for self-harm, depression, attention-deficit hyperactivity disorder, ASD, alcohol use and schizophrenia increased more in students than in non-students over time. Older students experienced greater risk of self-harm and mental health conditions, whereas younger students were more at risk of alcohol use than non-student counterparts.

**Conclusions:**

Mental health conditions in students are common and diverse. While at university, students require person-centred stepped care, integrated with local third-sector and healthcare services to address specific conditions.

There are growing concerns about the rise of self-harm and mental health problems among young people in the UK and worldwide.^1^ There are a number of suggested explanations for this rise, ranging from social media use and financial adversity to increased academic pressure and the prevalence inflation hypothesis.^2^ Whatever the reason (and it is likely to be a complex interplay of factors), as access to higher education widens, the UK's student population more closely reflects the country's wider socioeconomic and demographic make-up. Increasing numbers of young people from disadvantaged backgrounds in higher education may bring increased risks of certain conditions compared with previous cohorts, and the prevalence of self-harm and mental health conditions could become increasingly similar between students and non-students.^1^ These two issues, increased rates generally and widening participation, may be partly reflected in the five-fold increase in the number of students who disclosed a mental health condition to their institution over the past decade.^3^ However, it is worth noting that the rate of suicide among university students is considerably lower than in the general population.^4^

The financial and academic stressors of university life itself have also changed and may have adversely affected students’ mental health. Current students are faced with larger debts than previous generations (affected by tuition fees, costs of living, loss of maintenance grants) when they are managing their own finances for the first time. These sorts of socioeconomic factors, as well as living alone, being female and being part of an ethnic minority group, have all been associated with poorer mental health in university students.^1,4,5^ Although the transition to university may coincide with a high-risk period for the onset of psychopathology^6^ and a loss of pre-existing social support networks,^7^ evidence suggests that the onset of psychopathology typically starts prior to university entry.^5,8^ The consequences of mental health conditions for university students include poorer engagement, academic performance and attainment, with increased levels of substance use and drop-out.^8,9^

No previous studies have examined students’ trajectories for mental health conditions and health-related outcomes using routinely collected data before and during university, over time and compared with non-students. We aimed to evaluate the association between self-harm, neurodevelopmental disorders and mental health conditions and (a) becoming a university student (versus non-student), (b) university drop-out and (c) incidence in university students and non-students using novel linkage between primary and secondary routinely collected healthcare data and higher education students’ data covering the population of Wales. Our analyses also included the examination of being a student (versus non-student) as a moderator variable and of exposure variables such as sex, deprivation, age at study entry, higher education provider, academic year and study year.

## Method

**Fig. 1:**
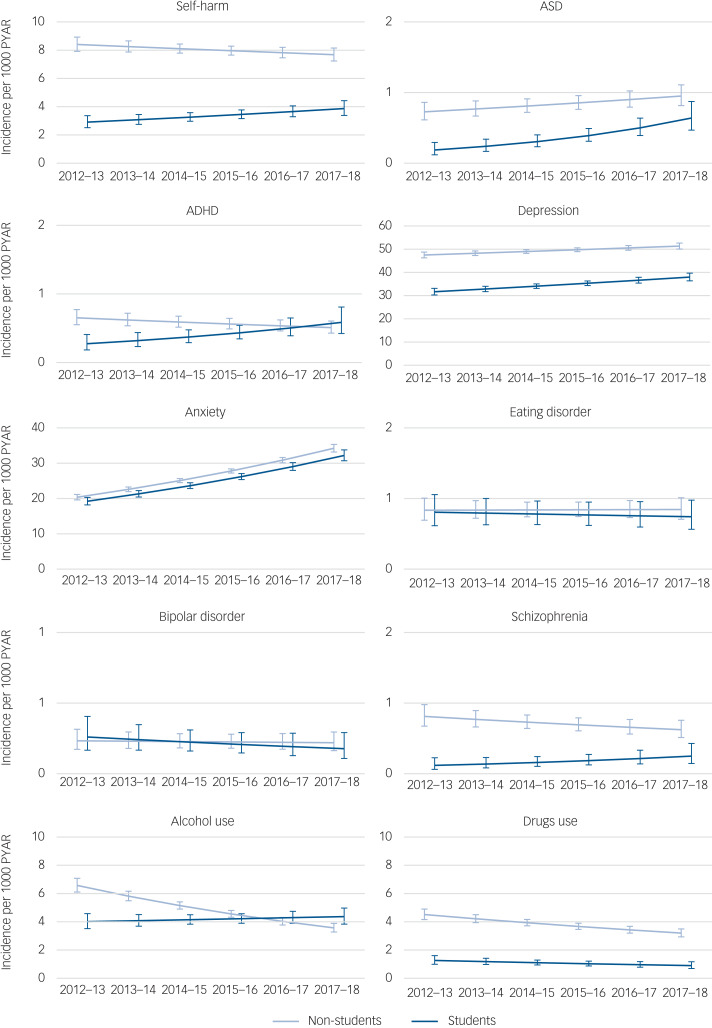
Post-estimation with marginal means for self-harm, neurodevelopmental disorders and mental health conditions for students and non-students, adjusting (averaging based on proportions) for sex, deprivation gradient, age at entry, study years, self-harm and mental health diagnoses before the index date. Vertical axes represent incidence (per 1000 person-years at risk (PYAR) of self-harm and mental disorders for students and non-students. Horizontal axes represent time per academic year. ASD, autism spectrum disorder; ADHD, attention-deficit hyperactivity disorder.

This was a retrospective cohort study using electronic records for the population of Wales born between 1987 and 1999 (Fig. 1). Ethical approval was granted by the Secure Anonymised Information Linkage (SAIL) databank's Information Governance Review Panel, an independent body consisting of a range of government, regulatory and professional agencies, in line with ethical permissions already granted to the analysis of data in the SAIL Databank – approval number 1031.

### Data sources

We gathered students’ information from the Higher Education Statistics Agency (HESA), the designated data body for England, and statutory for Wales, Scotland and Northern Ireland to curate data about higher education. We linked HESA data from 2012–2013 to 2017–2018^10^ within the SAIL Databank (www.saildatabank.com), a repository of routinely collected health and administrative data for the population of Wales.^11,12^ We used the following SAIL data-sets: Welsh Demographic Service Dataset (demographics of people registered with a general practice (GP) in Wales); the Office for National Statistics deaths register; the Welsh Index of Multiple Deprivation 2011;^13^ the Welsh Longitudinal General Practice Database (attendance and clinical information for all GP interactions covering 86% of the population of Wales); and the Patient Episode Database for Wales (hospital in-patient, out-patient and daycare activity in Wales).

### Data linkage

The data linkage between HESA and other data-sets within the SAIL databank for each individual was performed by using anonymised linkage identifiers (anonymised linkage field (ALF)) provided by Digital Health and Care Wales, a trusted third-party organisation mandated to hold personally identifiable data. ALFs are created using deterministic linkage based on National Health Service (NHS) number, or probabilistic matching using available demographics (name, surname, date of birth, recorded sex, address) based on Welsh Demographic Service datasets.^11,12^ In the present study, we used all records linked deterministically or probabilistically with a linkage score >0.9.

### Student cohort

The student cohort included first-time undergraduates between the academic years 2012–2013 and 2017–2018, aged 18 to 24 years (born between 1987 and 1999), who at the date of university entry had Welsh residency and were registered with a GP providing data to SAIL in the preceding year. Open University students were excluded because of differences in mode of study and sociodemographic characteristics.^14^

For each individual, the index date was the date of entry to university. Follow-up time was from whichever was the latest of (a) date of birth, (b) start of registration with a GP providing data to SAIL and (c) start of registration in Welsh residency to whichever was the earliest of (a) the end of registration at a university (regardless of the reason for leaving), (b) end of registration in a Welsh residency, (c) end of registration with a GP providing data to SAIL, (d) death or (e) end of study period.

### Non-student cohort

The control group consisted of Welsh residents not enrolled at university. This started with all individuals born between 1987 and 1999 not included in the HESA data-set. Index dates and follow-up end dates were pseudo-randomly generated to mirror the distributions of age at entry and duration of follow-up of the student cohort. Individuals were then pseudo-randomly selected to ensure that the distribution of the year of birth of the comparison group mirrored that of the student cohort.^15^ This two-step selection process for the control cohort allowed us to compare cohorts with similar age, index date and follow-up time characteristics and then adjust for sex and deprivation in the analysis where relevant. Non-students also needed to be living in Wales and registered with a GP providing data to SAIL 1 year before the index date.

### Measures and variables

#### Outcome variables

##### Student status

Student status was included as a binary variable (0 for non-students, 1 for students). This was used (a) as an outcome variable, (b) as a filtering variable (student-only subset) and (c) as an exposure and moderator, depending on analyses (further details below).

##### Drop-out from university

Among university students only, the drop-out variable was defined as a binary variable based on the reason for ending, obtained from HESA (further details at: https://www.hesa.ac.uk/collection/c17051/a/rsnend).

##### Self-harm and mental health conditions

We queried primary care and hospital admission data-sets to identify self-harm, neurodevelopmental disorders (autism spectrum disorder (ASD); attention-deficit hyperactivity disorder (ADHD)) and mental health conditions (depression, anxiety, eating disorder, bipolar disorder, schizophrenia, alcohol use, drug use) lifetime before and during university studies (or the pseudo-randomly generated study period for non-students). We used validated Read code (version 2) lists and algorithms^16^ in primary care and ICD-10 lists^17^ in secondary care from a previous study.^18^ This was completed using algorithms from the Adolescent Mental Health Data Platform (https://adolescentmentalhealth.uk), with their definition, method of development, any external validation and comprehensive code lists (Read codes and ICD-10 codes), and references openly accessible in the Concept Library (https://conceptlibrary.saildatabank.com/ADP). Further details are available in Supplementary Table 1 available at https://doi.org/10.1192/bjp.2024.90.

#### Exposure variables

Sex was entered as a binary variable. Age at index date (first entry to university for students, or pseudo-randomly generated date for non-students) in years was computed as a continuous variable. The academic year was entered as a continuous variable (from 1 to 6) representing the year (from 2012–2013 to 2017–2018). The study year was defined as a categorical variable, including first year, second year, third year and more than three years accounting for the time between the index date and end of follow-up. Among university students only, the higher education provider was included as a binary variable (1 for a provider in Wales, 0 for a provider not in Wales). We used Welsh Index of Multiple Deprivation (an official measure of small-area – approximately 1500 individuals – deprivation in Wales) quintiles as a categorical variable from 1 (least deprived) to 5 (most deprived). For each mental health condition, ‘event before’ was defined as a binary variable accounting for the presence of the outcome (self-harm or a mental health condition) before the index date.

### Analysis

Data were queried within the SAIL Databank using IBM DB2 9.7 SQL. All statistical analyses were conducted in R (version 4.1.1) with R-Studio (version 2022.07.0+) in Windows 10, with the level of significance set at *P* = 0.05 and with 95% CI. We used descriptive statistics and binomial probabilities with Wilson continuity correction^19^ to summarise the characteristics of the students and non-students. We complied with HESA's privacy-protecting rounding methodology where all counts of people were rounded to the nearest multiple of five and averages and percentages of groups less than 22.5 persons were excluded.^20^

We used logistic regression with robust standard error to calculate the odds ratios of being university students (versus non-students), accounting for sex (male versus female), deprivation, self-harm, neurodevelopmental disorders and mental health conditions happening during the lifetime before the index date. Subsequently, we used logistic regression with robust standard error to calculate the odds ratios of drop-out (versus non-drop-out) among university students only, accounting for higher education provider (Welsh versus non-Welsh), sex (male versus female), age at entry to university, deprivation, self-harm, neurodevelopmental disorders and mental health conditions diagnosed during the lifetime before the index date, and self-harm, neurodevelopmental disorders and mental health condition diagnosed during university.

We used Poisson regression with robust standard errors to calculate incidence rate ratios (IRR) for self-harm and mental health conditions accounting for academic year (from 2012–2013 to 2017–2018), sex (male, female), age at entry, study year (first, second, third and more than three), self-harm, neurodevelopmental disorders and mental health conditions happening during their lifetime before the index date. For each outcome variable, incidence events were defined as first events at the end of a 12-month period. Unadjusted incidence rates were computed for each outcome, using person-years at risk (PYAR) as the denominator. Student status was used as a second-order interaction factor in all analyses. We also compared students versus non-students, age at entry and academic year using post-estimations with marginal means.

We computed unadjusted prevalence of self-harm, neurodevelopmental disorders and mental health conditions of students and non-students at the index date (Supplementary Table 2). We conducted several sensitivity analyses. The first was to ascertain potential differences when defining incidence as the first life-time event instead of using a 12-month period (Supplementary Table 3). Further analyses were conducted separately using academic year and age at entry as categorical, rather than continuous, variables (Supplementary Tables 4 and 5) and yielded similar results.

## Results

In total 95 760 students and 151 795 non-students were included in the study cohort (see Supplementary Fig. 1 for the study flow diagram; and Table 1 for demographics).

**Table 1:** Demographic characteristics of students and non-students

Characteristic	Students, *n* (% [95% CI]) *n* = 96 760 (38.9% [38.7–39.1%])	Non-students, *n* (% [95% CI]) *n* = 151 795 (61.1% [60.9–61.2%])
**Sex**		
Male	42 515 (43.9% [43.6–44.2%])	83 390 (54.9% [54.6–55.2%])
Female	54 250 (56.0% [55.7–56.4%])	68 405 (45.0% [44.8–45.3%])
**Deprivation**		
1 (least deprived)	29 320 (30.3% [30.0–30.6%])	22 500 (14.8% [14.6–15.0%)
2	19 330 (20.0% [19.7–20.2%])	22 860 (15.0% [14.8%15.2%])
3	18 895 (19.5% [19.2–19.7%])	28 925 (19.0% [18.8–19.2%])
4	16 155 (16.6% [16.4–16.9%])	34 590 (22.8% [22.6–23.0%])
5 (most deprived)	13 060 (13.5% [13.3–13.7%])	42 920 (28.2% [28.0–28.5%])
**Academic year**		
2012–2013	36 020 (37.2% [36.2–37.5%])	57 770 (38.0% [37.8–38.3%])
2013–2014	35 810 (37.0% [36.7–37.3%])	59 805 (39.3% [39.1–39.6%])
2014–2015	35 400 (36.5% [36.2–36.8%])	60 495 (39.8% [39.6–40.0%])
2015–2016	36 235 (37.4% [37.1–37.7%])	64 510 (42.4% [42.2–42.7%])
2016–2017	36 800 (38.0% [37.7–38.3%])	64 385 (42.4% [42.1–42.6%])
2017–2018	36 435 (37.6% [37.3–37.9%])	63 685 (42.0% [41.8–42.3%])
**Age at entry**		
18 years	37 465 (38.7% [38.4–39.0%])	58 480 (38.5% [38.2–38.7%])
19 years	34 725 (37.0% [36.7–37.3%])	54 045 (35.6% [35.3–35.8%])
20 years	12 470 (12.8% [12.6–13.1%])	19 725 (12.9% [12.8–13.1%)
21 years	4965 (5.1% [4.9–5.3%])	8170 (5.3% [5.2–5.4%])
22 years	3195 (3.3% [3.2–3.4%])	5130 (4.0% [3.9–4.1%])
23 years	2250 (2.3% [2.2–2.4%])	3670 (2.4% [2.3–2.5%])
24 years	1690 (1.7% [1.6–1.8%])	2580 (1.7% [1.6–1.8%])
**Year of study**		
First	72 190 (74.6% [74.3–74.8%])	113 270 (74.6% [74.4–74.8%])
Second	63 680 (65.8% [65.5–66.1%])	103 055 (67.8% [67.6–68.1%])
Third	57 515 (59.4%% [59.1–59.7%])	97 413 (64.1% [63.9–34.4%])
>3	23 300 (24.0% [23.8–24.3%])	56 905 (37.4% [37.2–37.7%])
**Comorbidities**^a^		
0	90 155 (93.2% [93.0–93.3%])	133 585 (88.0% [87.8–88.2%])
1	5660 (5.8% [5.7–6.0%])	14 545 (9.5% [9.4–9.7%])
2	820 (0.8% [0.8–0.9%])	2950 (1.9% [1.9–2.0%])
3+	125 (0.1% [0.1–0.1%])	715 (0.5% [0.4–0.5%])
**Higher education provider**		
In Wales	55 910 (57.8% [57.5–58.1%])	
Not in Wales	40 580 (42.2% [41.9–42.5%])	
**Drop-out from university**		
Yes	10 095 (10.4% [10.2–10.6%])	
No	86 665 (89.6% [89.4–89.8%])	
**Reason for drop-out (*n* = 10 095)**		
Academic failure/left in bad standing/not permitted to progress	3005 (29.8% [28.9–30.7%])	
Health reasons	525 (5.2% [4.8–5.7%])	
Death	25 (0.2% [0.2–0.4%])	
Financial reasons	155 (1.5% [1.3–1.8%])	
Other personal reasons & dropped out	2840 (28.1% [27.3–29.0%])	
Written off after lapse of time	735 (7.3% [6.8–7.8%])	
Exclusion	390 (3.9% [3.5–4.3%])	
Gone into employment	420 (4.2% [3.8–4.6%])	
Other	2000 (19.8% [19.0–20.6%])	

a. Comorbidities include: self-harm, autism spectrum disorder, attention-deficit hyperactivity disorder, depression, anxiety, eating disorder, bipolar disorder, schizophrenia, alcohol use and drugs use.

### Association between self-harm, neurodevelopmental disorders and mental health conditions and becoming a university student

The first analysis considered the association between self-harm, neurodevelopmental disorders and mental health conditions and becoming a university student versus a non-student (Table 2). The results of the logistic regression showed that females had higher odds of becoming a university student compared with males. Higher levels of deprivation resulted in lower odds of becoming a student. Individuals who had a record of self-harm, neurodevelopmental disorder, depression, schizophrenia, or alcohol or drug use before the index date had lower odds of becoming university students. Anxiety, eating disorder and bipolar disorder before the index date were not related to the odds of becoming student.

**Table 2:** Logistic regression (robust standard error) computing the odds ratios of being a university student (versus non-student)^a^

	Students		
Predictors	Odds ratio	95% CI	*P*
Sex (female)	1.63	1.60–1.65	**<0.001**
Deprivation quintile 2	0.65	0.63–0.67	**<0.001**
Deprivation quintile 3	0.50	0.49–0.52	**<0.001**
Deprivation quintile 4	0.37	0.36–0.37	**<0.001**
Deprivation quintile 5 (most deprived)	0.24	0.23–0.25	**<0.001**
Self-harm before	0.53	0.51–0.56	**<0.001**
ASD before	0.54	0.49–0.59	**<0.001**
ADHD before	0.24	0.22–0.26	**<0.001**
Depression before	0.69	0.66–0.71	**<0.001**
Anxiety before	1.03	0.99–1.07	0.129
Eating disorder before	1.01	0.94–1.09	0.722
Bipolar disorder before	1.18	0.82–1.71	0.368
Schizophrenia before	0.57	0.40–0.79	**0.001**
Alcohol before	0.60	0.56–0.64	**<0.001**
Drugs before	0.31	0.27–0.36	**<0.001**
Observations	248 555		
Tjur's *R*^2^	0.086		

ASD, autism spectrum disorder; ADHD, attention-deficit hyperactivity disorder.

a. Adjusted for sex (male *v.* female), deprivation, self-harm, neurodevelopmental disorders, and mental health conditions happening before the index date.

### Association between self-harm, neurodevelopmental disorders, and mental health conditions and drop-out among university students

The second analysis considered factors associated with drop-out (versus no drop-out) among students only. The results of the logistic regression showed that students who were female or enrolled with a higher education provider in Wales (versus higher education provider not in Wales), were at lower risk of drop-out from university. Higher levels of deprivation and an older age at entry to university resulted in higher odds of drop-out. Students who had a record of self-harm, ADHD, depression, anxiety or alcohol use before entering university were at higher odds of drop-out from university. Students with an event of self-harm, depression, schizophrenia and drug use during their studies were also at higher risk of drop-out, whereas students with an event of anxiety during their studies were at lower risk of drop-out (Table 3).

**Table 3:** Logistic regression (robust standard error) computing the odds ratios of drop-out among university students^a^

	Drop-out from university		
Predictors	Odds ratios	95% CI	*P*
Higher education provider (in Wales)	0.93	0.89–0.97	**0.001**
Sex (female)	0.69	0.66–0.72	**<0.001**
Deprivation quintile 2	1.20	1.13–1.28	**<0.001**
Deprivation quintile 3	1.23	1.15–1.30	**<0.001**
Deprivation quintile 4	1.37	1.28–1.46	**<0.001**
Deprivation quintile 5 (most deprived)	1.44	1.34–1.54	**<0.001**
Age at entry	1.10	1.08–1.11	**<0.001**
Self-harm before	1.32	1.17–1.49	**<0.001**
ASD before	0.90	0.70–1.17	0.442
ADHD before	1.31	1.01–1.70	0.046
Depression before	1.34	1.23–1.46	**<0.001**
Anxiety before	1.17	1.07–1.27	**<0.001**
Eating disorder before	1.13	0.95–1.34	0.174
Bipolar disorder before	1.40	0.69–2.86	0.355
Schizophrenia before	1.14	0.54–2.39	0.729
Alcohol before	1.43	1.24–1.66	**<0.001**
Drugs before	0.93	0.66–1.33	0.708
Self-harm	1.70	1.38–2.09	**<0.001**
ASD	0.71	0.36–1.39	0.319
ADHD	1.05	0.66–1.68	0.834
Depression	1.15	1.05–1.24	**0.001**
Anxiety	0.71	0.64–0.79	**<0.001**
Eating disorder	0.87	0.58–1.32	0.515
Bipolar disorder	0.77	0.38–1.58	0.479
Schizophrenia	3.07	1.55–6.11	**0.001**
Alcohol	0.92	0.72–1.16	0.468
Drugs	1.71	1.20–2.44	**0.003**
Observations	96 760		
Tjur's *R*^2^	0.011		

ASD, autism spectrum disorder; ADHD, attention-deficit hyperactivity disorder.

a. Adjusting for higher education in Wales (versus not in Wales), sex (male versus female), age at entry at university, deprivation and mental health conditions happening before the index date and during university.

### Incidence of self-harm and mental health conditions between university students and non-students

The third set of analyses compared the incidence of self-harm, neurodevelopmental disorders and mental health conditions during university (or a similar time period for non-students) between university students and non-students in Wales. Crude incidence rates were lower for students compared with non-students for all the studied conditions, except anxiety (higher for students) and eating disorders (non-significant) (Supplementary Table 6).

The Poisson models can be seen in Table 4. Post-estimation with marginal means in the adjusted models showed that incidence rates were lower for students compared with non-students for self-harm, ASD, depression, anxiety, schizophrenia and drugs use (Supplementary Fig. 2).

**Table 4:** (Part 1) Poisson model for self-harm, neurodevelopmental disorders and mental health conditions accounting for student status (no/yes), academic years, sex (male/female), deprivation, age at entry, study year, comorbidities and event before the index date

	Self-harm			ASD			ADHD			Depression			Anxiety		
Predictors	IRR	95% CI	*P*	IRR	95% CI	*P*	IRR	95% CI	*P*	IRR	95% CI	*P*	IRR	95% CI	*P*
[student]	0.03	0.01–0.07	**<0.001**	0.00	0.00–0.01	**<0.001**	0.01	0.00–0.09	**<0.001**	0.09	0.06–0.12	**<0.001**	0.23	0.15–0.35	**<0.001**
Academic year	0.98	0.96–1.00	0.058	1.06	1.01–1.11	**0.024**	0.95	0.91–0.99	**0.029**	1.02	1.01–1.02	**<0.001**	1.11	1.10–1.12	**<0.001**
Sex (female)	0.94	0.88–1.01	0.076	0.87	0.72–1.05	0.15	0.84	0.70–1.01	0.064	1.85	1.79–1.90	**<0.001**	1.74	1.68–1.81	**<0.001**
Deprivation [2]^a^	1.19	1.04–1.35	**0.012**	1.28	0.95–1.72	0.108	1.26	0.93–1.70	0.135	1.05	0.99–1.11	0.079	1.00	0.93–1.07	0.995
Deprivation [3]	1.29	1.14–1.46	**<0.001**	1.38	1.05–1.82	**0.021**	1.15	0.86–1.53	0.34	1.11	1.06–1.17	**<0.001**	1.05	0.98–1.12	0.181
Deprivation [4]	1.31	1.17–1.48	**<0.001**	1.17	0.89–1.53	0.262	1.19	0.91–1.56	0.205	1.23	1.17–1.29	**<0.001**	1.11	1.04–1.18	**0.001**
Deprivation [5]	1.54	1.37–1.72	**<0.001**	1.27	0.97–1.65	0.08	1.45	1.12–1.88	**0.004**	1.39	1.33–1.46	**<0.001**	1.10	1.04–1.17	**0.002**
Age at entry	0.86	0.83–0.88	**<0.001**	0.82	0.76–0.88	**<0.001**	0.86	0.80–0.91	**<0.001**	0.95	0.94–0.96	**<0.001**	0.96	0.95–0.98	**<0.001**
Study year [second]	0.96	0.89–1.04	0.357	1.01	0.84–1.22	0.876	1.19	1.00–1.42	0.056	1.14	1.10–1.18	**<0.001**	1.10	1.05–1.15	**<0.001**
Study year [third]	0.89	0.82–0.96	**0.004**	0.82	0.67–1.01	0.068	1.12	0.93–1.35	0.237	1.21	1.17–1.26	**<0.001**	1.19	1.13–1.25	**<0.001**
Study year [>3]	0.75	0.68–0.84	**<0.001**	0.86	0.66–1.12	0.272	0.80	0.61–1.03	0.086	1.25	1.20–1.31	**<0.001**	1.22	1.15–1.29	**<0.001**
Self-harm before	3.53	3.24–3.84	**<0.001**	1.62	1.26–2.07	**<0.001**	1.47	1.19–1.81	**<0.001**	1.67	1.60–1.74	**<0.001**	1.32	1.25–1.40	**<0.001**
ASD before	0.97	0.78–1.20	0.776	55.55	45.87–67.28	**<0.001**	1.19	0.94–1.51	0.147	0.84	0.75–0.94	**0.002**	1.23	1.09–1.38	**0.001**
ADHD before	1.75	1.55–1.98	**<0.001**	1.69	1.36–2.10	**<0.001**	95.36	79.44–114.48	**<0.001**	1.46	1.36–1.56	**<0.001**	1.23	1.12–1.35	**<0.001**
Depression before	2.64	2.44–2.86	**<0.001**	1.70	1.39–2.08	**<0.001**	1.57	1.30–1.89	**<0.001**	3.77	3.65–3.89	**<0.001**	1.99	1.90–2.07	**<0.001**
Anxiety before	1.39	1.27–1.52	**<0.001**	1.46	1.19–1.81	**<0.001**	1.27	1.04–1.56	**0.022**	1.58	1.53–1.65	**<0.001**	3.41	3.27–3.56	**<0.001**
Eating disorder before	1.48	1.26–1.73	**<0.001**	1.80	1.25–2.59	**0.002**	0.90	0.56–1.43	0.644	1.20	1.11–1.30	**<0.001**	1.24	1.13–1.37	**<0.001**
Bipolar before	1.24	0.81–1.91	0.323	1.60	0.68–3.75	0.283	0.98	0.31–3.15	0.977	0.68	0.50–0.92	**0.014**	1.16	0.85–1.59	0.351
Schizophrenia before	1.75	1.33–2.31	**<0.001**	1.84	0.91–3.70	0.09	1.00	0.53–1.87	0.992	1.21	0.99–1.47	0.061	1.55	1.24–1.93	**<0.001**
Alcohol before	1.79	1.61–1.98	**<0.001**	0.64	0.39–1.04	0.069	1.03	0.79–1.34	0.822	1.20	1.13–1.27	**<0.001**	1.15	1.07–1.24	**<0.001**
Drugs before	1.65	1.45–1.87	**<0.001**	0.67	0.34–1.32	0.247	1.54	1.17–2.03	**0.002**	1.43	1.32–1.54	**<0.001**	1.50	1.36–1.65	**<0.001**
[student] × academic year	1.08	1.03–1.13	**0.002**	1.21	1.07–1.37	**0.002**	1.22	1.07–1.39	**0.002**	1.02	1.00–1.04	**0.014**	1.00	0.98–1.02	0.937
[student] × sex (female)	1.74	1.46–2.08	**<0.001**	0.54	0.35–0.85	**0.008**	0.87	0.57–1.31	0.498	0.82	0.78–0.87	**<0.001**	1.00	0.93–1.07	0.974
[student] × deprivation [2]	0.90	0.70–1.17	0.427	1.11	0.55–2.24	0.781	0.85	0.45–1.59	0.603	0.91	0.83–0.99	**0.034**	0.99	0.89–1.10	0.878
[student] × deprivation [3]	0.79	0.61–1.02	0.068	1.24	0.63–2.43	0.526	0.85	0.45–1.61	0.619	0.90	0.82–0.98	**0.014**	0.92	0.83–1.02	0.132
[student] × deprivation [4]	0.77	0.59–1.00	**0.049**	2.10	1.10–4.01	**0.024**	0.92	0.49–1.75	0.806	0.90	0.82–0.98	**0.016**	0.88	0.79–0.98	**0.018**
[student] × deprivation [5]	0.84	0.65–1.09	0.185	1.40	0.69–2.85	0.349	0.67	0.35–1.31	0.243	0.79	0.72–0.86	**<0.001**	0.95	0.85–1.05	0.307
[student] × age at entry	1.14	1.08–1.20	**<0.001**	1.33	1.16–1.53	**<0.001**	1.23	1.07–1.41	**0.003**	1.12	1.10–1.14	**<0.001**	1.08	1.05–1.10	**<0.001**
[student] × study [second]	0.94	0.78–1.15	0.561	1.02	0.62–1.67	0.948	1.99	1.17–3.40	**0.012**	1.16	1.08–1.24	**<0.001**	1.07	0.99–1.17	0.098
[student] × study [third]	0.79	0.63–0.98	**0.036**	1.02	0.58–1.80	0.932	1.16	0.62–2.18	0.645	1.10	1.02–1.18	**0.012**	1.1	1.01–1.20	**0.035**
[student] × study [>3]	1.29	0.96–1.73	0.088	1.41	0.68–2.91	0.351	5.31	2.80–10.09	**<0.001**	1.45	1.32–1.60	**<0.001**	1.27	1.14–1.43	**<0.001**
Observations	539 905			540 750			539 115			521 995			536 635		
Nagelkerke's *R*^2^	0.097			0.237			0.305			0.099			0.072		

IRR, incidence rate ratio; ASD, autism spectrum disorder; ADHD, attention-deficit hyperactivity disorder.

a. Deprivation quintiles [2]–[5], where [5] is the most deprived.

**Table 4:** (Part 2) Poisson model for self-harm, neurodevelopmental disorders and mental health conditions accounting for student status (no/yes), academic years, sex (male/female), deprivation, age at entry, study year, comorbidities and event before the index date

	Eating disorder			Bipolar disorder			Schizophrenia			Alcohol			Drugs		
Predictors	IRR	95% CI	*P*	IRR	95% CI	*P*	IRR	95% CI	*P*	IRR	95% CI	*P*	IRR	95% CI	*P*
[student]	0.09	0.01–0.79	**0.03**	0.05	0.00–1.64	0.093	0.00	0.00–0.00	**<0.001**	0.38	0.11–1.27	0.117	0.01	0.00–0.06	**<0.001**
Academic year	1.00	0.95–1.06	0.929	0.99	0.91–1.07	0.762	0.95	0.89–1.01	0.086	0.88	0.86–0.91	**<0.001**	0.93	0.91–0.96	**<0.001**
Sex (female)	3.07	2.44–3.86	**<0.001**	1.69	1.21–2.36	**0.002**	0.48	0.38–0.60	**<0.001**	0.72	0.65–0.78	**<0.001**	0.42	0.38–0.46	**<0.001**
Deprivation 2^a^	1.06	0.76–1.48	0.73	1.10	0.63–1.94	0.737	0.77	0.51–1.15	0.2	1.26	1.06–1.49	**0.01**	1.27	1.06–1.53	**0.01**
Deprivation [3]	0.85	0.61–1.18	0.337	0.89	0.51–1.53	0.665	1.09	0.76–1.55	0.635	1.24	1.05–1.47	**0.009**	1.27	1.07–1.52	**0.006**
Deprivation [4]	0.87	0.63–1.19	0.383	1.20	0.73–1.98	0.48	0.84	0.59–1.19	0.318	1.31	1.12–1.54	**0.001**	1.33	1.13–1.57	**0.001**
Deprivation [5]	0.91	0.68–1.23	0.547	1.21	0.75–1.96	0.43	1.08	0.77–1.50	0.658	1.35	1.16–1.57	**<0.001**	1.56	1.33–1.83	**<0.001**
Age at entry	0.83	0.76–0.89	**<0.001**	0.93	0.84–1.03	0.167	0.85	0.78–0.92	**<0.001**	0.91	0.88–0.95	**<0.001**	0.94	0.91–0.97	**<0.001**
Study year [second]	1.13	0.90–1.42	0.282	1.32	0.90–1.94	0.158	1.14	0.88–1.48	0.329	0.96	0.86–1.07	0.454	1.13	1.01–1.26	**0.034**
Study year [third]	0.96	0.75–1.24	0.762	1.54	1.04–2.28	**0.03**	1.15	0.87–1.50	0.327	0.92	0.83–1.03	0.166	1.04	0.92–1.17	0.525
Study year [>3]	0.69	0.49–0.97	**0.031**	2.49	1.65–3.77	**<0.001**	1.52	1.12–2.05	**0.007**	0.87	0.75–1.00	0.053	1.32	1.16–1.51	**<0.001**
Self-harm before	1.72	1.35–2.19	**<0.001**	3.43	2.51–4.68	**<0.001**	1.77	1.26–2.47	**0.001**	2.21	1.95–2.51	**<0.001**	2.41	2.11–2.76	**<0.001**
ASD before	1.51	0.84–2.69	0.169	1.44	0.69–2.98	0.329	0.69	0.37–1.28	0.24	0.67	0.48–0.93	**0.018**	0.61	0.44–0.86	**0.004**
ADHD before	0.84	0.48–1.45	0.532	2.66	1.73–4.09	**<0.001**	1.62	1.12–2.33	**0.01**	1.55	1.31–1.83	**<0.001**	1.84	1.58–2.13	**<0.001**
Depression before	1.85	1.51–2.26	**<0.001**	5.57	4.03–7.71	**<0.001**	2.53	1.94–3.30	**<0.001**	1.59	1.42–1.77	**<0.001**	2.30	2.05–2.57	**<0.001**
Anxiety before	1.76	1.42–2.17	**<0.001**	2.22	1.64–3.00	**<0.001**	1.12	0.82–1.54	0.47	1.38	1.22–1.57	**<0.001**	1.42	1.25–1.62	**<0.001**
Eating disorder before	10.34	8.48–12.61	**<0.001**	1.11	0.63–1.94	0.716	1.46	0.81–2.64	0.211	1.45	1.15–1.83	**0.002**	0.87	0.63–1.19	0.375
Bipolar before	0.86	0.21–3.55	0.83	33.77	20.73–55.01	**<0.001**	0.99	0.44–2.21	0.98	1.40	0.74–2.66	0.306	1.17	0.62–2.21	0.629
Schizophrenia before	0.48	0.06–3.61	0.478	1.45	0.60–3.50	0.403	44.25	29.15–67.16	**<0.001**	1.37	0.91–2.07	0.131	2.24	1.66–3.02	**<0.001**
Alcohol before	1.00	0.69–1.46	0.986	1.46	0.98–2.19	0.065	0.88	0.59–1.30	0.519	2.73	2.38–3.13	**<0.001**	1.65	1.42–1.92	**<0.001**
Drugs before	1.30	0.79–2.15	0.307	0.87	0.50–1.54	0.644	3.98	2.64–5.99	**<0.001**	2.10	1.76–2.51	**<0.001**	4.94	4.21–5.79	**<0.001**
[student] × academic year	0.98	0.90–1.07	0.683	0.94	0.80–1.10	0.434	1.22	1.02–1.47	**0.029**	1.15	1.10–1.21	**<0.001**	1.00	0.92–1.09	0.989
[student] × sex (female)	1.87	1.19–2.94	**0.007**	1.00	0.53–1.88	0.996	1.31	0.70–2.47	0.402	1.01	0.85–1.19	0.943	0.93	0.70–1.25	0.648
[student] × deprivation [2]	0.84	0.53–1.34	0.476	0.67	0.27–1.68	0.393	0.98	0.31–3.06	0.973	1.00	0.76–1.31	0.989	1.43	0.92–2.22	0.115
[student] × deprivation [3]	0.76	0.46–1.24	0.271	1.13	0.48–2.68	0.776	0.92	0.33–2.60	0.881	0.86	0.66–1.14	0.3	1.08	0.68–1.71	0.737
[student] × deprivation [4]	0.93	0.58–1.49	0.758	0.50	0.20–1.29	0.151	1.98	0.77–5.04	0.154	0.91	0.69–1.19	0.491	1.29	0.82–2.03	0.264
[student] × deprivation [5]	0.71	0.43–1.18	0.185	0.77	0.33–1.80	0.543	1.60	0.61–4.18	0.336	0.99	0.75–1.30	0.948	0.87	0.53–1.42	0.579
[student] × age at entry	1.12	1.00–1.26	**0.041**	1.18	1.00–1.40	**0.049**	1.43	1.20–1.70	**<0.001**	1.03	0.97–1.09	0.371	1.18	1.09–1.28	**<0.001**
[student] × study [second]	0.96	0.66–1.39	0.819	1.13	0.53–2.42	0.743	2.84	1.07–7.54	**0.036**	0.80	0.66–0.99	**0.037**	0.70	0.49–1.01	0.056
[student] × study [third]	1.23	0.83–1.82	0.306	1.23	0.57–2.63	0.594	2.41	0.85–6.81	0.098	0.69	0.55–0.87	**0.001**	0.83	0.57–1.21	0.341
[student] × study [>3]	1.65	0.93–2.93	0.086	1.62	0.70–3.73	0.258	4.95	1.77–13.88	**0.002**	0.98	0.73–1.32	0.895	1.08	0.69–1.68	0.748
Observations	540 705			540 795			540 755			540 530			540 440		
Nagelkerke's *R*^2^	0.087			0.188			0.172			0.041			0.119		

IRR, incidence rate ratio; ASD, autism spectrum disorder; ADHD, attention-deficit hyperactivity disorder.

a. Deprivation quintile [2]–[5], where [5] is the most deprived.

### Sex

The incidence of self-harm was higher for females compared with males in the student population, and with similar gradients among non-students. The incidence of depression was higher for females among non-students, but with lower gradients among students. This means the higher rate of depression among females is reduced among university students, compared with non-students. The incidence of ASD was lower for females in students only, whereas incidence of ADHD, anxiety, bipolar disorder, schizophrenia, alcohol and drugs use showed similar sex gradients between students and non-students.

### Academic year

Interactions between student status and academic year for each condition are shown in Fig. 1. Incidence of anxiety increased with time (between the academic years 2012–2013 and 2017–2018) at a similar rate among students and non-students. The incidence of self-harm and schizophrenia increased with time among students only. The incidence of ASD and depression increased with time among non-students, and with a higher rate among students. The incidence of ADHD and alcohol use increased with time for students and decreased for non-students. The incidence of drug use decreased with time among non-students, and with a similar rate among students (Table 4).

### Age at entry

**Fig. 2:**
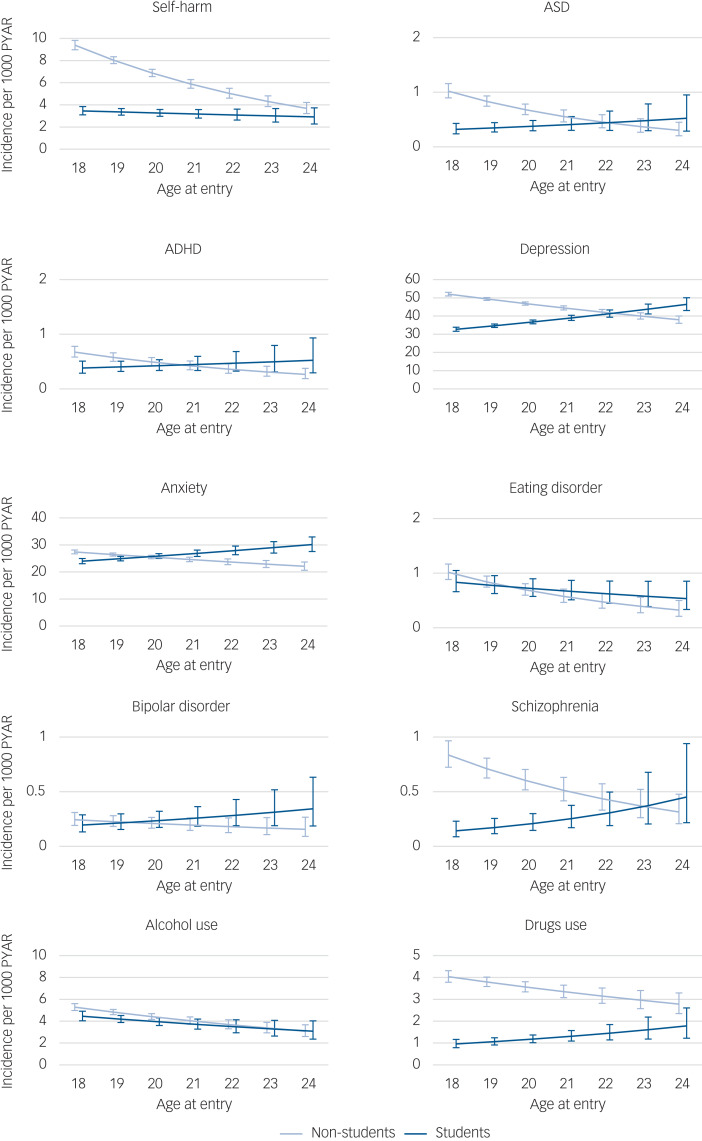
Post-estimation with marginal means for self-harm, neurodevelopmental disorders, and mental health conditions for students and non-students adjusting (averaging based on proportions) for academic year, sex, deprivation gradient, study years, self-harm and mental health diagnoses before the index date. Vertical axes represent incidence (per 1000 person-years at risk (PYAR)) of self-harm and mental disorders for students and non-students. Horizontal axes represent age at entry (in years). ASD, autism spectrum disorder; ADHD, attention-deficit hyperactivity disorder.

Interactions between student status and age at entry for each condition are shown in Fig. 2. The incidence of anxiety increased with age at a higher rate among students than among non-students. Incidence of depression, ADHD and schizophrenia increased with age among non-students only. Incidence of ASD and self-harm decreased with age among non-students but increased with age among students. Incidence of eating disorder, bipolar disorder, alcohol and drug use varied with age equally among non-students and students (Table 4).

### Study year

The incidence of depression and anxiety increased with study year (second, third and more than three versus first year) at higher rate among students than non-students. The incidence of ADHD increased with study year among students only, and the incidence of alcohol use decreased with study year among students only (Table 4).

## Discussion

This study linked person-level routinely collected data and included 96 760 students and 151 795 non-students. Overall, our results showed that being male, self-harm and mental health conditions recorded before university entry, and higher deprivation levels, resulted in lower odds of becoming a student and higher odds of drop-out from university among those who do become students. Self-harm, depression, anxiety, ASD, drug use and schizophrenia were lower for students compared with non-students with similar characteristics. However, IRRs for self-harm, depression, ADHD, ASD, alcohol use and schizophrenia increased more in students than non-students over time. Furthermore, students entering university at a later age experienced greater risk of self-harm and mental health conditions, whereas students entering university at an earlier age and first year students were more at risk of alcohol use than non-student counterparts.

The results of our study generally support previous evidence showing that university students have similar^1,21^ or better mental health than non-students.^22^ However, we also show that such relative risks vary greatly across conditions. Furthermore, and in line with recent evidence from two population based-cohorts in England,^23^ our results showed increased self-harm, neurodevelopmental disorders and mental health conditions among students, with differences between students and non-students gradually converging over time.

Certain conditions, such as self-harm, ASD, ADHD, depression, schizophrenia, and alcohol or drug use, are negatively related to the odds of becoming a university student – particularly ADHD. This could directly relate to the consequences of the condition itself but also to established associations with disruptions in education, such as higher rates of school absences and exclusions affecting engagement and attainment.^18^

Students with a record of self-harm, ADHD, depression, anxiety and alcohol use before the index date were more likely to drop out of university. Students with a record of self-harm, depression, schizophrenia and drug use during their studies were also more likely to drop out, whereas students with a record of anxiety during their studies were less likely to drop out. The variety and complexity of students’ mental health problems before and during university influence their academic outcomes,^24^ and that students arrive at university with a wide range of conditions already affecting their ability to cope requiring dedicated high-intensity support.^25^ Campus-based well-being resources and advisory and peer support such as self-help resources are likely not sufficient to attend to the variety and range of students’ needs.^25,26^ Our results demonstrate that Welsh students studying outside of Wales were also more likely to drop out. Similarly to international students, additional interventions may be required to support the transition of these students from further education and on attendance at universities outside of Wales.^27^

Although some conditions, such as anxiety, eating disorders or drug use, demonstrate trends similar to those of the general population, university students also have mental health needs and trajectories that differ from those of the general population. Our ability to detect such differences likely lies in our analysis at scale. For instance, our results show that the incidence of self-harm, neurodevelopmental disorders, depression, schizophrenia and alcohol use have particularly increased in the student population over time, although for neurodevelopmental disorders this may reflect better access to screening and diagnostic services. This further demonstrates the need for an integrated stepped-care approach within universities, including counselling and supported referral pathways to mental health services when required.^6^

It is well understood that the age at onset of certain mental health conditions coincides with time at university,^28^ but our results demonstrate overall an increased risk of self-harm, neurodevelopmental disorders and mental health conditions for students entering university at a later age. For depression and anxiety, this may be associated with stigma and financial pressures, or more difficult integration within the student community owing to the age gap.^29,30^ Therefore, we recommend universities not only provide support to first year students but also to carefully monitor and support the mental health of their older students.

The increase in alcohol use among students but not non-students over time is in line with existing evidence.^31^ Some studies suggest that alcohol use among university students could be related to financial concerns.^29,30^ However, our results show that this increase is widespread and not related to deprivation levels, with younger individuals and particularly first year students at higher risk of alcohol use. It may reflect a drinking culture influenced more by the sociocultural context than by individuals’ characteristics.^32,33^ Targeted brief evidence-based interventions are therefore required to reduce the increase in alcohol use among university students, particularly among younger and first year students.

Overall, we need to understand what risk and exacerbating factors are contributing to the development of mental health problems in students and what can be done to address systemic issues and make the university environment promote mental health in students.

In this respect, our results are in line with the calls for scaling up and integrated mental health services for university students.^6,34,35^ The breadth of students’ mental health needs requires the development and integration of a multidisciplinary person-centred stepped-care approach within universities.^6,35^ Integrated systems for student mental health could include triage and access to well-being support for transient conditions and situational difficulties such as isolation and loneliness. On-campus mental health multidisciplinary teams including counselling, augmented where appropriate with in-reach psychology and psychiatry, and supported transition for those with pre-existing conditions and referral pathways to mental health services when required is needed.^6^ This whole-system approach should include education and training in mental health literacy and suicide awareness for both students and staff to help identify signs of illness and distress and signposting to appropriate resources and services.^34^

Eventually, and despite policies and activities to widen the access to university,^36^ our results show a clear stratification in the odds of becoming a university student, with people from the most deprived areas and those with previous mental health conditions having less chance of accessing university than healthy individuals from less deprived areas. Although the deprivation gradient is strongly related to depression among non-students, this is not the case for students. Therefore, while deprivation hinders the chance of becoming student, once at university, students from more deprived areas do not have higher risk of depression than their more privileged peers.

### Strengths and limitations

By linking person-level routinely collected data covering a whole population we were able to meaningfully compare self-harm, neurodevelopmental disorders and mental health conditions between university students and non-students while accounting for sex, deprivation, age at entry, study year and time in 96 760 undergraduate students and 151 795 non-students. This provided a unique understanding of key commonalities and differences in mental health conditions between students and non-students and the size of the study allowed us to include less common conditions such as schizophrenia.

Secondary analysis of routinely collected data not specifically collected for research has inherent limitations: recorded sex but not gender; poorly recorded ethnicity; potential misclassification bias; unmeasured confounding or missing data.^37^ We attended to these issues carefully, for instance by using validated code lists to identify outcomes of interest. Certain mental health conditions (e.g. bipolar disorder, schizophrenia) are rare and their results should be interpreted carefully. Furthermore, our results showed that certain conditions increased the odds of university drop-out among students, whereas other conditions (e.g. bipolar disorder, eating disorder) did not. Further research is needed to clarify these differences.

We did not account for academic performance among students, which will be a focus of future research. In this study, we decided to compute the age at entry to university and the year of study at which each mental health condition occurred. Therefore, we could not include the actual age when each mental health condition happened, because of multicollinearity issues. Another limitation is that we were only able to include students with primary and secondary health records in Wales. This meant we did not include the pre-university history of international students or UK students registered elsewhere; or those who are not registered with a GP in Wales during university; or those who do not seek help from services, or where this is not recognised or recorded. Just over half of students in Wales's universities come from Wales and two in five undergraduates from Wales study in England.^38^ We were unable to ascertain any systematic differences between those students who are registered with a GP and those who are not. Further research is also required to explore possible converging trends in incidence of self-harm, neurodevelopmental disorders and mental health conditions between students and non-students over time. Further work is also required to better understand the increase in the incidence of self-harm, neurodevelopmental disorders, schizophrenia and alcohol use among students over time.

### Conclusions

Mental health conditions in students are common, diverse, wide-ranging and they influence the academic outcomes of university students. University students have mental health needs and trajectories that differ from those of the general population. This diversity and complexity of students’ needs requires an integrated person-centred stepped-care approach within universities that includes triage, access to well-being support, counselling and referral pathways to mental health services.^6^

## Supporting information

John et al. supplementary material 1 John et al. supplementary material

John et al. supplementary material 2 John et al. supplementary material

John et al. supplementary material 3 John et al. supplementary material

John et al. supplementary material 4 John et al. supplementary material

John et al. supplementary material 5 John et al. supplementary material

John et al. supplementary material 6 John et al. supplementary material

John et al. supplementary material 7 John et al. supplementary material

John et al. supplementary material 8 John et al. supplementary material

## Data Availability

The data used in this study are available in the Secure Anonymised Information Linkage (SAIL) Databank at Swansea University (Swansea, UK) via the Adolescent Mental Health Data Platform, but as restrictions apply they are not publicly available. All proposals to use SAIL data are subject to review by an independent Information Governance Review Panel (IGRP). Before any data can be accessed, approval must be given by the IGRP. The IGRP gives careful consideration to each project to ensure proper and appropriate use of SAIL data. When access has been granted, it is gained through a privacy-protecting safe haven and remote access system referred to as the SAIL Gateway. SAIL has established an application process to be followed by anyone who would like to access data via SAIL at https://saildatabank.com/data/apply-to-work-with-the-data/.
