# Supplementary figures and images for: Trends in incidence of self-harm, neurodevelopmental and mental health conditions among university students compared with the general population: nationwide electronic data linkage study in Wales
Source: Br J Psychiatry. 2024 Sep;225(3):389–400. doi: 10.1192/bjp.2024.90 (PMC11536190; doi:10.1192/bjp.2024.90)

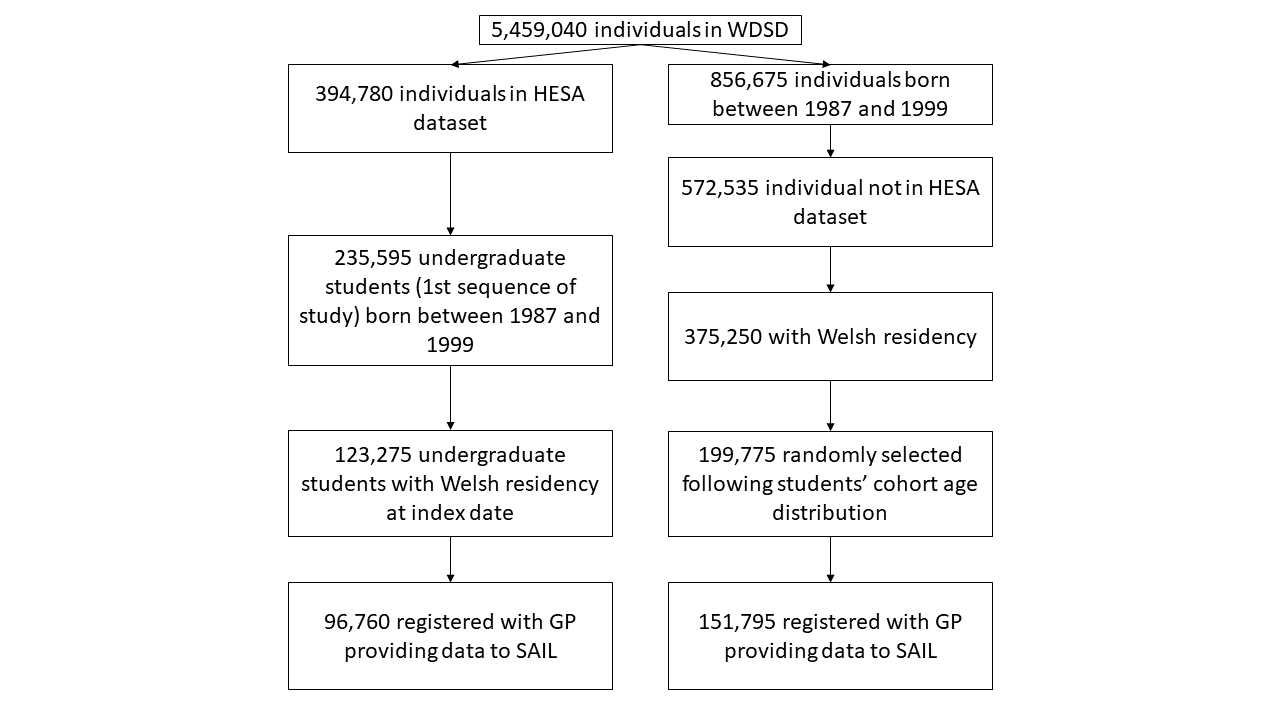


Figure 1 – Flow diagram of cohort selection

Supplement: John et al. supplementary material 1 — John et al. supplementary material [file S0007125024000904sup001.docx]
