# Supplementary material for: Trends in incidence of self-harm, neurodevelopmental and mental health conditions among university students compared with the general population: nationwide electronic data linkage study in Wales
Source: Br J Psychiatry. 2024 Sep;225(3):389–400. doi: 10.1192/bjp.2024.90 (PMC11536190; doi:10.1192/bjp.2024.90)
Supplement: John et al. supplementary material 2 — John et al. supplementary material [file S0007125024000904sup002.docx]

Figure 2 - Postestimation with marginal means for self-harm, ND, and mental health conditions for students and non-students adjusting (averaging based on proportions) for sex, deprivation gradient, age at entry, study years, self-harm and mental health diagnoses before the index date. Y-axes represent incidence (per 1000 PYAR) with 95% CI of self-harm and mental disorders for students and non-students. X-axes self-harm and mental health conditions. * depicts a significant difference (p < 0.05) between students and non-students
