# Supplementary material for: Trends in incidence of self-harm, neurodevelopmental and mental health conditions among university students compared with the general population: nationwide electronic data linkage study in Wales
Source: Br J Psychiatry. 2024 Sep;225(3):389–400. doi: 10.1192/bjp.2024.90 (PMC11536190; doi:10.1192/bjp.2024.90)
Supplement: John et al. supplementary material 3 — John et al. supplementary material [file S0007125024000904sup003.docx]

Supplementary table 1 - Phenotypes of events of self-harm, neurodevelopmental disorders, and mental health

| Phenotype | Definition | Source |
| --- | --- | --- |
| *Self-harm* | Self-harm is defined as non-fatal intentional self-poisoning or self-injury, regardless of motivation or intention. Events of poisoning and overdose with analgesics, although commonly implicated in suicidal behaviours, were not included as they are often accidental. We identify self-harm using all the concepts below **in conjunction**. These code lists are based on validated, published and comparable lists ([Marchant et. al. 2020](https://adc.bmj.com/content/105/4/347), [Thomas et. al. 2014](https://bpspubs.onlinelibrary.wiley.com/doi/10.1111/bcp.12059)). | https://conceptlibrary.saildatabank.com/phenotypes/PH936/detail/ |
| *ADHD* | Attention-Deficit hyperactivity disorder (ADHD) neurodevelopmental condition with secondary behavioural, social, and educational difficulties. ADHD is defined as a behavioural syndrome characterized by the core symptoms of hyperactivity, impulsivity, and inattention which can lead to psychological, social, education, or occupational impairment. ADHD can be identified with Read Codes (primary care) and/or ICD-10 codes. Read codes and ICD-10 codes have been taken from previously validated code lists (John et al, 2022) and include diagnoses and prescriptions. | https://conceptlibrary.saildatabank.com/phenotypes/PH932/detail/ |
| *ASD* | Autism spectrum disorder (ASD) refers any one of a group of disorders with an onset typically occurring during the preschool years and characterized by difficulties with social communication and social interaction and restricted and repetitive patterns in behaviours, interests, and activities. The term “spectrum” is used because of the heterogeneity in the presentation and severity of ASD symptoms, as well as in the skills and level of functioning of individuals who have ASD.  Autism Spectrum Disorder (ASD) can be identified with Read Codes (primary care) and/or ICD-10 codes. Codes to identify Autism Spectrum Disorder (ASD) come from Underwood et al. (2022) and are selecting for the maximum sensitivity and specificity of autistic phenotypes. | https://conceptlibrary.saildatabank.com/phenotypes/PH933/detail/ |
| *Learning difficulties* | Learning difficulties is a broad term used for different conditions that affect the normal learning ability of individuals (Burt, 2004, p. 786). Learning difficulties can be identified with Read Codes (primary care) and/or ICD-10 codes collated from previously validated code lists or were compiled in collaboration with clinicians (John et al, 2022). | https://conceptlibrary.saildatabank.com/phenotypes/PH935/detail/ |
| *Conduct disorder* | Conduct disorders are characterised by a persistent and repetitive pattern of behaviour where age-appropriate societal norms or rules, or the rights of others are violated. These codes have been taken from previously validates code lists (John et al, 2019, John et al, 2022).  Conduct disorders can be identified with Read Codes (primary care) and/or ICD-10 codes. | https://conceptlibrary.saildatabank.com/phenotypes/PH934/detail/ |
| *Depression* | Depression can be identified with Read Codes (primary care) and/or ICD-10 codes.  This concept is made up of a number of sub-concepts. Code lists for each of these concepts are based on validated published and comparable lists [John et. el. 2016; Cornish et al 2016; John et. al., 2022] and reviewed by a clinician in the study team (Prof Ann John).  The core diagnosis codes are essential for identifying depression. Depression diagnoses can be identified in both Read Codes and ICD-10. Other sub-concepts apply only to Read Codes and can be included in combination as necessary dependent on study design. Various combinations of code lists have been previously described and validated utilising the MHI-5 as the gold standard (John et al., 2016) | https://conceptlibrary.saildatabank.com/phenotypes/PH1114/detail/ |
| *Anxiety* | Anxiety can be identified with Read Codes (primary care) and/or ICD-10 codes.  This concept is made up of a number of sub-concepts. Code lists for each of these concepts are based on validated published and comparable lists [John et. el. 2016; Cornish et al 2016; John et. al., 2022] and reviewed by a clinician in the study team (Prof Ann John).  The core diagnosis codes are essential for identifying anxiety and can be identified using both Read Codes or ICD-10 codes. The remaining sub-concepts are found in Read Codes only and can be included in combination as necessary dependent on study design. Various combinations of code lists have been previously described and validated utilising the MHI-5 as the gold standard (John et al., 2016). | https://conceptlibrary.saildatabank.com/phenotypes/PH1113/detail/ |
| *Eating disorder* | This list was developed from previously validated code lists (Micali et al., 2013) that used weight, height and body mass index to validate 10% of records. These previously validated codes were further developed and refined with clinician input including an eating disorder specialist.  This definition of eating disorders encompasses: anorexia nervosa (AN), bulimia nervosa (BN) and eating disorder not otherwise specified (EDNOS). EDNOS generally is recorded for an eating disorder which causes distress and impairment but does not meet the full criteria for the other subtypes. We included atypical AN and atypical BN in the category EDNOS. Atypical AN and atypical BN have commonalities with AN and BN, but they do not meet the threshold for diagnosis. Other non-specified eating disorders, such as binge- eating disorder (Micali et al., 2013) are also categorised as EDNOS. | https://conceptlibrary.saildatabank.com/phenotypes/PH1116/detail/ |
| *Bipolar disorder* | Bipolar disorder is a mental health condition that affects individuals' moods, which can swing from one extreme (depression) to another (mania). The following code list includes Read codes and ICD-10 codes for bipolar disorder and other mood related disorders used in the following papers (John et. al. 2018; John et. al. 2022) and reviewed by a clinician in the study (Prof Ann John). | https://conceptlibrary.saildatabank.com/ADP/phenotypes/PH937/detail/ |
| *Schizophrenia* | Schizophrenia is a long-term mental health condition. It causes a range of different psychological symptoms such as hallucinations and delusions. The following code list includes Read codes and ICD-10 codes for schizophrenia-related disorders that include schizophrenia, schizotypal and delusional disorders used in the following papers (John et. al. 2018; John et. al. 2022) and reviewed by a clinician in the study (Prof Ann John). | https://conceptlibrary.saildatabank.com/ADP/phenotypes/PH939/detail/ |
| *Other psychotic disorder* | Psychotic disorders and severe mental illnesses that are not included as schizophrenia or bipolar disorder are categorised as Other psychotic disorders. The following code list includes Read codes and ICD-10 codes for other psychotic disorders used in the following papers (John et. al. 2018; John et. al. 2022) and reviewed by a clinician in the study (Prof Ann John). Read codes for 'other psychotic disorders' that were mapped to the ICD-10 codes for acute and transient psychotic disorders (F23), depressive episodes/disorders with psychotic symptoms (F32.3 and F33.3) were added to the list as reviewed by two clinicians (Prof. Keith Lloyd and Prof. Ann John) in the study team. | https://conceptlibrary.saildatabank.com/ADP/phenotypes/PH938//detail/ |
| *Alcohol misuse* | Codes denoting alcohol use, including diagnoses, symptoms, observations, medications, behaviours, referrals and contacts with other services. This list contains prescriptions relevant to treatment for alcohol use disorder (disulfiram and acamprosate) as almost all recipients were found to have a history of alcohol use (Rees et. al., 2022); and excludes those used primarily for pain management. Codes requiring an associated value of units relating to alcohol consumption volumes are excluded, as these do not confidently denote alcohol use disorder on their own.  These code lists are based on validated, published and comparable lists (Rees et. al., 2022; DelPozo-Banos et. al., 2018; Kim et. al., 2017; NHS, 2018; NHS, 2019; Syed et. al., 2018; Walsh et. al., 2018) and reviewed by a clinician in the study team (Prof Ann John). | https://conceptlibrary.saildatabank.com/phenotypes/PH1107/detail/ |
| *Drugs misuse* | Codes denoting drug use, including diagnoses, symptoms, observations, medications, behaviours (e.g., “injecting drug user”), referrals and contacts with other services. It includes codes for illegal drugs but excludes tobacco, in keeping with the literature. This list contains prescriptions relevant to treatment for drug use disorder (naltrexone, lofexidine and methadone) as almost all recipients were found to have a history of drug use (Rees et. al., 2022); and excludes those used primarily for pain management. For buprenorphine, the list includes only prescriptions where 90% or more recipients had a history of drug use disorder, as assessed during validation (Rees et. al., 2022).  These code lists are based on validated, published and comparable lists (Rees et. al., 2022; DelPozo-Banos et. al., 2018; NHS, 2018; NHS, 2019; Syed et. al., 2018; Walsh et. al., 2018) and reviewed by a clinician in the study team (Prof Ann John). | https://conceptlibrary.saildatabank.com/phenotypes/PH1108/detail/ |
