# Supplementary material for: Trends in incidence of self-harm, neurodevelopmental and mental health conditions among university students compared with the general population: nationwide electronic data linkage study in Wales
Source: Br J Psychiatry. 2024 Sep;225(3):389–400. doi: 10.1192/bjp.2024.90 (PMC11536190; doi:10.1192/bjp.2024.90)
Supplement: John et al. supplementary material 4 — John et al. supplementary material [file S0007125024000904sup004.docx]

Supplementary table 2 – Unadjusted prevalence of self-harm, neurodevelopmental disorders, and mental health conditions of students and non-students at university entry (or pseudo-random index date for non-students).

| ***CHARACTERISTIC*** | **STUDENTS (PERC. [95% CI])  N = 96,760 (38·9% [38·7%-39·1%])** | **NON-STUDENTS (PERC. [95% CI])  N = 151,795 (61·1% [60·9%-61·2%])** |
| --- | --- | --- |
| *Self-harm* | 2,450 (2.5% [2.4%-2.6%]) | 9,380 (6.2% [6.1%-6.3%]) |
| *ASD* | 615 (0.6% [0.6%-0.7%]) | 2,540 (1.7% [1.6%-1.7%]) |
| *ADHD* | 500 (0.5% [0.5%-0.7%]) | 4,725 (3.1% [3.0%-3.2%]) |
| *Depression* | 6,785 (7.0% [6.9%-7.2%]) | 16,975 (11.0% [10.9%-11.2%]) |
| *Anxiety* | 5,875 (6.1% [5.9%-6.2%]) | 10,285 (6.8% [6.7%-6.9%]) |
| *Bipolar disorder* | 50 (<0.1% [0.0%-0.1%]) | 155 (0.1% [0.1%-0.1%]) |
| *Schizophrenia* | 50 (<0.1% [0.0%-0.1%])) | 310 (0.2% [0.2%-0.2%]) |
| *Eating disorder* | 1,295 (1.3% [1.3%-1.4%]) | 2,130 (1.4% [1.3%-1.5%]) |
| *Drugs misuse* | 255 (0.3% [0.2%-0.3%]) | 2,575 (1.7% [1.6%-1.8%]) |
| *Alcohol misuse* | 1,425 (1.5% [1.4%-1.6%]) | 5,210 (3.4% [3.4%-3.6%]) |
