# Supplementary material for: Trends in incidence of self-harm, neurodevelopmental and mental health conditions among university students compared with the general population: nationwide electronic data linkage study in Wales
Source: Br J Psychiatry. 2024 Sep;225(3):389–400. doi: 10.1192/bjp.2024.90 (PMC11536190; doi:10.1192/bjp.2024.90)
Supplement: John et al. supplementary material 5 — John et al. supplementary material [file S0007125024000904sup005.docx]

Supplementary table 3 (part 1) Poisson model for lifetime incidence of self-harm, neurodevelopmental disorders and mental health conditions accounting for student status (No/Yes), Academic years, sex (Male/Female), deprivation quintile age at entry and year of study. IRR = incidence rate ratio.

|  | **SELF-HARM** | | | **ASD** | | | **ADHD** | | | **DEPRESSION** | | | **ANXIETY** | | |
| --- | --- | --- | --- | --- | --- | --- | --- | --- | --- | --- | --- | --- | --- | --- | --- |
| *Predictors* | *IRR* | *CI* | *p* | *IRR* | *CI* | *p* | *IRR* | *CI* | *p* | *IRR* | *CI* | *p* | *IRR* | *CI* | *p* |
| [student] | 0.03 | 0.01 – 0.10 | **<0.001** | 0 | 0.00 – 0.01 | **<0.001** | 0 | 0.00 – 0.02 | **<0.001** | 0.08 | 0.05 – 0.13 | **<0.001** | 0.16 | 0.09 – 0.27 | **<0.001** |
| Academic year | 1.02 | 1.00 – 1.04 | 0.091 | 1.22 | 1.14 – 1.31 | **<0.001** | 1.16 | 1.06 – 1.27 | **0.001** | 1.09 | 1.08 – 1.10 | **<0.001** | 1.16 | 1.14 – 1.17 | **<0.001** |
| Sex (female) | 1.09 | 1.01 – 1.18 | **0.025** | 0.6 | 0.47 – 0.76 | **<0.001** | 0.63 | 0.46 – 0.86 | **0.003** | 1.93 | 1.86 – 2.00 | **<0.001** | 2 | 1.91 – 2.09 | **<0.001** |
| Deprivation [2] | 1.21 | 1.02 – 1.42 | **0.024** | 1.11 | 0.73 – 1.69 | 0.625 | 1.36 | 0.78 – 2.37 | 0.282 | 1.12 | 1.05 – 1.21 | **0.002** | 0.99 | 0.91 – 1.08 | 0.833 |
| Deprivation [3] | 1.42 | 1.22 – 1.65 | **<0.001** | 1.04 | 0.69 – 1.56 | 0.853 | 1.1 | 0.63 – 1.92 | 0.732 | 1.19 | 1.11 – 1.27 | **<0.001** | 1.1 | 1.02 – 1.20 | **0.017** |
| Deprivation [4] | 1.5 | 1.30 – 1.73 | **<0.001** | 1.09 | 0.74 – 1.60 | 0.67 | 1 | 0.58 – 1.73 | 0.986 | 1.33 | 1.25 – 1.42 | **<0.001** | 1.21 | 1.12 – 1.31 | **<0.001** |
| Deprivation [5] | 1.76 | 1.53 – 2.02 | **<0.001** | 0.94 | 0.64 – 1.38 | 0.742 | 1.39 | 0.84 – 2.28 | 0.2 | 1.55 | 1.46 – 1.65 | **<0.001** | 1.21 | 1.12 – 1.30 | **<0.001** |
| Age at entry | 0.9 | 0.87 – 0.93 | **<0.001** | 0.79 | 0.70 – 0.88 | **<0.001** | 0.93 | 0.82 – 1.05 | 0.238 | 0.95 | 0.94 – 0.96 | **<0.001** | 0.99 | 0.98 – 1.01 | 0.46 |
| Study year [second] | 0.91 | 0.83 – 1.00 | 0.06 | 1.02 | 0.77 – 1.35 | 0.878 | 1.04 | 0.73 – 1.49 | 0.819 | 1.04 | 1.00 – 1.09 | **0.049** | 1.05 | 1.00 – 1.11 | 0.07 |
| Study year [third] | 0.75 | 0.67 – 0.83 | **<0.001** | 0.81 | 0.60 – 1.11 | 0.191 | 0.71 | 0.47 – 1.07 | 0.105 | 0.92 | 0.88 – 0.97 | **0.001** | 0.98 | 0.93 – 1.04 | 0.586 |
| Study year [>3] | 0.57 | 0.50 – 0.66 | **<0.001** | 0.58 | 0.38 – 0.87 | **0.009** | 0.56 | 0.33 – 0.96 | **0.034** | 0.85 | 0.80 – 0.90 | **<0.001** | 0.96 | 0.89 – 1.03 | 0.22 |
| [student] * academic year | 1.08 | 1.02 – 1.14 | **0.011** | 1.12 | 0.95 – 1.32 | 0.18 | 1.22 | 1.01 – 1.48 | **0.034** | 1.03 | 1.01 – 1.05 | **0.011** | 1.01 | 0.98 – 1.03 | 0.521 |
| [student] * sex (female) | 1.57 | 1.29 – 1.92 | **<0.001** | 0.64 | 0.35 – 1.17 | 0.149 | 0.83 | 0.47 – 1.47 | 0.521 | 0.82 | 0.77 – 0.88 | **<0.001** | 0.96 | 0.88 – 1.04 | 0.303 |
| [student] * Deprivation [2] | 0.88 | 0.65 – 1.18 | 0.38 | 1.54 | 0.57 – 4.16 | 0.398 | 0.81 | 0.34 – 1.91 | 0.63 | 0.81 | 0.72 – 0.91 | **<0.001** | 1 | 0.88 – 1.14 | 0.994 |
| [student] * Deprivation [3] | 0.78 | 0.58 – 1.04 | 0.089 | 2.27 | 0.89 – 5.74 | 0.084 | 0.61 | 0.24 – 1.55 | 0.299 | 0.84 | 0.75 – 0.94 | **0.002** | 0.87 | 0.77 – 0.98 | **0.027** |
| [student] * Deprivation [4] | 0.69 | 0.51 – 0.94 | **0.017** | 1.98 | 0.75 – 5.20 | 0.167 | 0.93 | 0.38 – 2.29 | 0.88 | 0.84 | 0.76 – 0.94 | **0.002** | 0.78 | 0.69 – 0.88 | **<0.001** |
| [student] * Deprivation [5] | 0.78 | 0.58 – 1.04 | 0.096 | 2.95 | 1.14 – 7.60 | **0.026** | 0.72 | 0.29 – 1.75 | 0.465 | 0.69 | 0.62 – 0.77 | **<0.001** | 0.89 | 0.78 – 1.01 | 0.066 |
| [student] * age at entry | 1.13 | 1.06 – 1.21 | **<0.001** | 1.42 | 1.15 – 1.75 | **0.001** | 1.37 | 1.14 – 1.65 | **0.001** | 1.12 | 1.10 – 1.15 | **<0.001** | 1.1 | 1.07 – 1.13 | **<0.001** |
| [student] * study [second] | 1.03 | 0.83 – 1.28 | 0.787 | 0.76 | 0.37 – 1.57 | 0.454 | 2.6 | 1.24 – 5.49 | **0.012** | 1.13 | 1.04 – 1.23 | **0.003** | 1.04 | 0.94 – 1.14 | 0.471 |
| [student] * study [third] | 0.84 | 0.65 – 1.08 | 0.17 | 1.15 | 0.55 – 2.39 | 0.71 | 2.05 | 0.85 – 4.91 | 0.108 | 1.09 | 1.00 – 1.19 | 0.062 | 1.07 | 0.96 – 1.18 | 0.22 |
| [student] * study [>3] | 1.35 | 0.94 – 1.95 | 0.102 | 1.94 | 0.72 – 5.21 | 0.189 | 8.79 | 3.55 – 21.80 | **<0.001** | 1.35 | 1.19 – 1.54 | **<0.001** | 1.19 | 1.04 – 1.38 | **0.014** |
| Observations | 509920 | | | 533495 | | | 528605 | | | 466425 | | | 492995 | | |
| R^2^ Nagelkerke | 0.021 | | | 0.03 | | | 0.024 | | | 0.025 | | | 0.022 | | |

Supplementary table 3 (part 2) Poisson model for lifetime incidence of self-harm, neurodevelopmental disorders and mental health conditions accounting for student status (No/Yes), Academic years, sex (Male/Female), deprivation quintile age at entry and year of study. IRR = incidence rate ratio.

|  | **EATING DISORDER** | | | **BIPOLAR DISORDER** | | | **SCHIZOPHRENIA** | | | **ALCOHOL** | | | **DRUGS** | | |
| --- | --- | --- | --- | --- | --- | --- | --- | --- | --- | --- | --- | --- | --- | --- | --- |
| *Predictors* | *IRR* | *CI* | *p* | *IRR* | *CI* | *p* | *IRR* | *CI* | *p* | *IRR* | *CI* | *p* | *IRR* | *CI* | *p* |
| [student] | 0.13 | 0.01 – 2.32 | 0.167 | 0.02 | 0.00 – 0.49 | **0.018** | 0 | 0.00 – 0.00 | **<0.001** | 0.15 | 0.04 – 0.56 | **0.005** | 0.01 | 0.00 – 0.04 | **<0.001** |
| Academic year | 1.06 | 1.00 – 1.13 | **0.046** | 1.03 | 0.94 – 1.13 | 0.529 | 0.99 | 0.92 – 1.07 | 0.819 | 0.9 | 0.88 – 0.93 | **<0.001** | 0.98 | 0.96 – 1.01 | 0.312 |
| Sex (female) | 3.25 | 2.57 – 4.12 | **<0.001** | 2.77 | 1.95 – 3.91 | **<0.001** | 0.51 | 0.39 – 0.66 | **<0.001** | 0.82 | 0.74 – 0.90 | **<0.001** | 0.47 | 0.42 – 0.52 | **<0.001** |
| Deprivation [2] | 1.26 | 0.84 – 1.88 | 0.261 | 1.26 | 0.65 – 2.47 | 0.493 | 0.79 | 0.49 – 1.28 | 0.345 | 1.38 | 1.14 – 1.67 | **0.001** | 1.31 | 1.07 – 1.61 | **0.009** |
| Deprivation [3] | 1.12 | 0.76 – 1.65 | 0.583 | 0.93 | 0.47 – 1.83 | 0.835 | 1.07 | 0.70 – 1.62 | 0.761 | 1.36 | 1.13 – 1.63 | **0.001** | 1.3 | 1.07 – 1.59 | **0.008** |
| Deprivation [4] | 0.98 | 0.67 – 1.44 | 0.928 | 1.47 | 0.81 – 2.68 | 0.205 | 0.96 | 0.64 – 1.46 | 0.863 | 1.45 | 1.22 – 1.73 | **<0.001** | 1.4 | 1.16 – 1.68 | **0.001** |
| Deprivation [5] | 1.2 | 0.84 – 1.72 | 0.308 | 1.87 | 1.06 – 3.30 | **0.03** | 1.39 | 0.95 – 2.02 | 0.089 | 1.52 | 1.28 – 1.80 | **<0.001** | 1.78 | 1.49 – 2.12 | **<0.001** |
| Age at entry | 0.86 | 0.79 – 0.95 | **0.001** | 1.09 | 0.97 – 1.21 | 0.141 | 0.89 | 0.80 – 0.98 | **0.019** | 0.92 | 0.89 – 0.96 | **<0.001** | 0.97 | 0.94 – 1.01 | 0.166 |
| Study year [second] | 1.12 | 0.87 – 1.44 | 0.363 | 1.2 | 0.78 – 1.83 | 0.414 | 1.17 | 0.88 – 1.56 | 0.288 | 0.93 | 0.83 – 1.04 | 0.223 | 1.09 | 0.97 – 1.24 | 0.155 |
| Study year [third] | 0.74 | 0.55 – 0.99 | **0.041** | 1.27 | 0.82 – 1.97 | 0.28 | 0.86 | 0.62 – 1.19 | 0.373 | 0.77 | 0.68 – 0.88 | **<0.001** | 0.88 | 0.76 – 1.00 | 0.058 |
| Study year [>3] | 0.52 | 0.35 – 0.78 | **0.002** | 1.49 | 0.92 – 2.43 | 0.108 | 0.91 | 0.62 – 1.34 | 0.641 | 0.66 | 0.55 – 0.78 | **<0.001** | 1.04 | 0.88 – 1.21 | 0.659 |
| [student] * academic year | 1 | 0.90 – 1.12 | 0.995 | 0.92 | 0.77 – 1.10 | 0.362 | 1.17 | 0.95 – 1.45 | 0.134 | 1.16 | 1.10 – 1.22 | **<0.001** | 0.99 | 0.90 – 1.09 | 0.886 |
| [student] * sex (female) | 2.07 | 1.23 – 3.49 | **0.006** | 0.73 | 0.38 – 1.39 | 0.334 | 1.24 | 0.62 – 2.51 | 0.541 | 0.88 | 0.74 – 1.06 | 0.18 | 0.93 | 0.67 – 1.28 | 0.641 |
| [student] * Deprivation [2] | 0.66 | 0.38 – 1.16 | 0.145 | 0.7 | 0.26 – 1.90 | 0.478 | 1.05 | 0.32 – 3.44 | 0.932 | 0.97 | 0.72 – 1.30 | 0.839 | 1.49 | 0.91 – 2.42 | 0.112 |
| [student] * Deprivation [3] | 0.55 | 0.31 – 0.99 | **0.046** | 1.17 | 0.44 – 3.12 | 0.756 | 1.08 | 0.37 – 3.16 | 0.889 | 0.83 | 0.61 – 1.11 | 0.206 | 1.19 | 0.72 – 1.98 | 0.493 |
| [student] * Deprivation [4] | 0.76 | 0.43 – 1.35 | 0.353 | 0.61 | 0.23 – 1.62 | 0.318 | 1.79 | 0.65 – 4.93 | 0.258 | 0.84 | 0.62 – 1.12 | 0.235 | 1.41 | 0.86 – 2.31 | 0.176 |
| [student] * Deprivation [5] | 0.64 | 0.36 – 1.13 | 0.122 | 0.56 | 0.21 – 1.48 | 0.243 | 0.98 | 0.33 – 2.90 | 0.968 | 0.92 | 0.68 – 1.23 | 0.566 | 0.78 | 0.45 – 1.36 | 0.382 |
| [student] * age at entry | 1.09 | 0.94 – 1.26 | 0.25 | 1.26 | 1.06 – 1.50 | **0.009** | 1.47 | 1.21 – 1.79 | **<0.001** | 1.08 | 1.01 – 1.16 | **0.025** | 1.2 | 1.10 – 1.32 | **<0.001** |
| [student] * study [second] | 0.89 | 0.58 – 1.36 | 0.589 | 1.13 | 0.50 – 2.55 | 0.774 | 2.71 | 0.94 – 7.83 | 0.066 | 0.77 | 0.62 – 0.95 | **0.014** | 0.83 | 0.57 – 1.22 | 0.345 |
| [student] * study [third] | 1.37 | 0.86 – 2.17 | 0.189 | 1.27 | 0.56 – 2.89 | 0.562 | 3.07 | 0.99 – 9.51 | 0.052 | 0.65 | 0.50 – 0.83 | **0.001** | 0.98 | 0.65 – 1.48 | 0.92 |
| [student] * study [>3] | 1.37 | 0.64 – 2.91 | 0.417 | 2.84 | 1.18 – 6.80 | **0.02** | 8.38 | 2.67 – 26.27 | **<0.001** | 1.02 | 0.72 – 1.43 | 0.914 | 1.18 | 0.70 – 1.98 | 0.544 |
| Observations | 532710 | | | 540205 | | | 539610 | | | 521750 | | | 531970 | | |
| R^2^ Nagelkerke | 0.031 | | | 0.025 | | | 0.031 | | | 0.009 | | | 0.038 | | |
