# Supplementary material for: Trends in incidence of self-harm, neurodevelopmental and mental health conditions among university students compared with the general population: nationwide electronic data linkage study in Wales
Source: Br J Psychiatry. 2024 Sep;225(3):389–400. doi: 10.1192/bjp.2024.90 (PMC11536190; doi:10.1192/bjp.2024.90)
Supplement: John et al. supplementary material 6 — John et al. supplementary material [file S0007125024000904sup006.docx]

Supplementary table 4 (part 1) Poisson model for mental health diagnoses accounting for student status (No/Yes), Academic years, sex (Male/Female), deprivation, age at entry (factor), study year, comorbidities, and event before. IRR = incidence rate ratio.

|  | **SELF-HARM** | | | **ASD** | | | **ADHD** | | | **DEPRESSION** | | | **ANXIETY** | | |
| --- | --- | --- | --- | --- | --- | --- | --- | --- | --- | --- | --- | --- | --- | --- | --- |
| *Predictors* | *IRR* | *CI* | *p* | *IRR* | *CI* | *p* | *IRR* | *CI* | *p* | *IRR* | *CI* | *p* | *IRR* | *CI* | *p* |
| [student] | 0.24 | 0.18 – 0.32 | **<0.001** | 0.17 | 0.07 – 0.39 | **<0.001** | 0.22 | 0.10 – 0.50 | **<0.001** | 0.63 | 0.57 – 0.70 | **<0.001** | 0.88 | 0.77 – 1.00 | **0.043** |
| Academic year | 0.98 | 0.96 – 1.00 | 0.057 | 1.06 | 1.01 – 1.11 | **0.025** | 0.95 | 0.91 – 1.00 | **0.029** | 1.02 | 1.01 – 1.02 | **<0.001** | 1.11 | 1.10 – 1.12 | **<0.001** |
| Sex (female) | 0.94 | 0.88 – 1.01 | 0.077 | 0.87 | 0.72 – 1.05 | 0.15 | 0.84 | 0.70 – 1.01 | 0.067 | 1.85 | 1.79 – 1.90 | **<0.001** | 1.74 | 1.68 – 1.81 | **<0.001** |
| Deprivation [2] | 1.19 | 1.04 – 1.35 | **0.012** | 1.28 | 0.95 – 1.72 | 0.105 | 1.26 | 0.93 – 1.71 | 0.131 | 1.05 | 0.99 – 1.11 | 0.082 | 1 | 0.93 – 1.07 | 0.998 |
| Deprivation [3] | 1.29 | 1.14 – 1.46 | **<0.001** | 1.39 | 1.05 – 1.82 | **0.02** | 1.15 | 0.87 – 1.54 | 0.328 | 1.11 | 1.06 – 1.17 | **<0.001** | 1.05 | 0.98 – 1.12 | 0.187 |
| Deprivation [4] | 1.31 | 1.16 – 1.48 | **<0.001** | 1.17 | 0.89 – 1.53 | 0.253 | 1.2 | 0.91 – 1.57 | 0.194 | 1.23 | 1.17 – 1.29 | **<0.001** | 1.11 | 1.04 – 1.18 | **0.001** |
| Deprivation [5] | 1.54 | 1.37 – 1.72 | **<0.001** | 1.26 | 0.97 – 1.65 | 0.081 | 1.46 | 1.13 – 1.90 | **0.004** | 1.39 | 1.33 – 1.46 | **<0.001** | 1.1 | 1.04 – 1.17 | **0.002** |
| age at entry [19] | 0.83 | 0.78 – 0.89 | **<0.001** | 0.92 | 0.78 – 1.09 | 0.323 | 0.8 | 0.68 – 0.94 | **0.005** | 0.99 | 0.96 – 1.02 | 0.523 | 0.96 | 0.92 – 1.00 | 0.052 |
| age at entry [20] | 0.75 | 0.68 – 0.83 | **<0.001** | 0.68 | 0.52 – 0.88 | **0.004** | 0.6 | 0.46 – 0.76 | **<0.001** | 0.92 | 0.88 – 0.96 | **<0.001** | 0.98 | 0.92 – 1.03 | 0.421 |
| age at entry [21] | 0.57 | 0.48 – 0.66 | **<0.001** | 0.47 | 0.29 – 0.77 | **0.002** | 0.72 | 0.52 – 1.01 | 0.054 | 0.88 | 0.82 – 0.93 | **<0.001** | 0.88 | 0.80 – 0.95 | **0.002** |
| age at entry [22] | 0.55 | 0.45 – 0.67 | **<0.001** | 0.56 | 0.31 – 1.00 | **0.049** | 0.39 | 0.22 – 0.68 | **0.001** | 0.85 | 0.79 – 0.92 | **<0.001** | 0.94 | 0.86 – 1.04 | 0.26 |
| age at entry [23] | 0.49 | 0.39 – 0.62 | **<0.001** | 0.23 | 0.09 – 0.62 | **0.004** | 0.64 | 0.37 – 1.11 | 0.112 | 0.78 | 0.71 – 0.86 | **<0.001** | 0.85 | 0.75 – 0.96 | **0.007** |
| age at entry [24] | 0.38 | 0.28 – 0.52 | **<0.001** | 0.31 | 0.10 – 0.96 | **0.043** | 0.51 | 0.24 – 1.07 | 0.075 | 0.66 | 0.59 – 0.73 | **<0.001** | 0.69 | 0.60 – 0.80 | **<0.001** |
| Study year [second] | 0.96 | 0.89 – 1.04 | 0.361 | 1.02 | 0.84 – 1.22 | 0.869 | 1.19 | 1.00 – 1.42 | 0.055 | 1.14 | 1.10 – 1.18 | **<0.001** | 1.1 | 1.05 – 1.15 | **<0.001** |
| Study year [third] | 0.89 | 0.82 – 0.96 | **0.005** | 0.82 | 0.67 – 1.02 | 0.07 | 1.12 | 0.93 – 1.35 | 0.24 | 1.21 | 1.17 – 1.26 | **<0.001** | 1.19 | 1.13 – 1.24 | **<0.001** |
| Study year [>3] | 0.75 | 0.68 – 0.84 | **<0.001** | 0.86 | 0.66 – 1.13 | 0.279 | 0.8 | 0.62 – 1.03 | 0.087 | 1.25 | 1.20 – 1.31 | **<0.001** | 1.22 | 1.15 – 1.29 | **<0.001** |
| Self-harm before | 3.53 | 3.24 – 3.84 | **<0.001** | 1.64 | 1.28 – 2.10 | **<0.001** | 1.46 | 1.18 – 1.80 | **<0.001** | 1.67 | 1.60 – 1.74 | **<0.001** | 1.32 | 1.25 – 1.40 | **<0.001** |
| ASD before | 0.97 | 0.78 – 1.20 | 0.761 | 55.47 | 45.80 – 67.19 | **<0.001** | 1.19 | 0.93 – 1.50 | 0.162 | 0.84 | 0.75 – 0.93 | **0.002** | 1.23 | 1.09 – 1.38 | **0.001** |
| ADHD before | 1.76 | 1.55 – 1.98 | **<0.001** | 1.69 | 1.36 – 2.10 | **<0.001** | 95.97 | 79.93 – 115.21 | **<0.001** | 1.46 | 1.36 – 1.56 | **<0.001** | 1.23 | 1.12 – 1.35 | **<0.001** |
| Depression before | 2.64 | 2.44 – 2.86 | **<0.001** | 1.7 | 1.39 – 2.08 | **<0.001** | 1.59 | 1.31 – 1.91 | **<0.001** | 3.76 | 3.64 – 3.88 | **<0.001** | 1.98 | 1.90 – 2.07 | **<0.001** |
| Anxiety before | 1.39 | 1.27 – 1.52 | **<0.001** | 1.46 | 1.19 – 1.81 | **<0.001** | 1.28 | 1.04 – 1.57 | **0.021** | 1.59 | 1.53 – 1.65 | **<0.001** | 3.41 | 3.27 – 3.56 | **<0.001** |
| Eating disorder before | 1.47 | 1.26 – 1.73 | **<0.001** | 1.8 | 1.25 – 2.58 | **0.002** | 0.89 | 0.55 – 1.42 | 0.614 | 1.2 | 1.11 – 1.30 | **<0.001** | 1.25 | 1.13 – 1.37 | **<0.001** |
| Bipolar before | 1.25 | 0.81 – 1.93 | 0.305 | 1.57 | 0.67 – 3.71 | 0.299 | 0.97 | 0.30 – 3.12 | 0.964 | 0.68 | 0.50 – 0.92 | **0.014** | 1.16 | 0.85 – 1.58 | 0.356 |
| Schizophrenia before | 1.75 | 1.33 – 2.31 | **<0.001** | 1.88 | 0.93 – 3.80 | 0.077 | 0.98 | 0.52 – 1.85 | 0.948 | 1.21 | 0.99 – 1.47 | 0.062 | 1.55 | 1.25 – 1.93 | **<0.001** |
| Alcohol before | 1.79 | 1.61 – 1.99 | **<0.001** | 0.63 | 0.38 – 1.03 | 0.063 | 1.03 | 0.79 – 1.34 | 0.833 | 1.2 | 1.13 – 1.27 | **<0.001** | 1.16 | 1.07 – 1.25 | **<0.001** |
| Drugs before | 1.65 | 1.45 – 1.88 | **<0.001** | 0.68 | 0.34 – 1.33 | 0.258 | 1.55 | 1.17 – 2.05 | **0.002** | 1.43 | 1.32 – 1.54 | **<0.001** | 1.5 | 1.36 – 1.65 | **<0.001** |
| [student] * academic year | 1.08 | 1.03 – 1.13 | **0.002** | 1.21 | 1.07 – 1.37 | **0.002** | 1.23 | 1.08 – 1.40 | **0.002** | 1.02 | 1.00 – 1.04 | **0.012** | 1 | 0.98 – 1.02 | 0.958 |
| [student] * sex (female) | 1.75 | 1.47 – 2.09 | **<0.001** | 0.54 | 0.35 – 0.85 | **0.008** | 0.87 | 0.57 – 1.33 | 0.523 | 0.83 | 0.78 – 0.88 | **<0.001** | 1 | 0.93 – 1.07 | 0.997 |
| [student] * Deprivation [2] | 0.91 | 0.70 – 1.17 | 0.45 | 1.05 | 0.52 – 2.14 | 0.885 | 0.86 | 0.46 – 1.61 | 0.634 | 0.91 | 0.83 – 0.99 | **0.034** | 0.99 | 0.89 – 1.10 | 0.861 |
| [student] * Deprivation [3] | 0.79 | 0.61 – 1.02 | 0.073 | 1.23 | 0.63 – 2.41 | 0.54 | 0.84 | 0.44 – 1.60 | 0.596 | 0.9 | 0.82 – 0.98 | **0.014** | 0.92 | 0.83 – 1.02 | 0.127 |
| [student] * Deprivation [4] | 0.77 | 0.59 – 0.99 | **0.044** | 2.09 | 1.09 – 3.98 | **0.025** | 0.9 | 0.47 – 1.71 | 0.751 | 0.9 | 0.82 – 0.98 | **0.014** | 0.88 | 0.79 – 0.98 | **0.015** |
| [student] * Deprivation [5] | 0.84 | 0.65 – 1.08 | 0.162 | 1.36 | 0.66 – 2.76 | 0.403 | 0.67 | 0.34 – 1.29 | 0.229 | 0.79 | 0.72 – 0.86 | **<0.001** | 0.94 | 0.85 – 1.05 | 0.277 |
| [student] * age at entry [19] | 1.26 | 1.04 – 1.54 | **0.018** | 0.87 | 0.52 – 1.48 | 0.613 | 1.06 | 0.64 – 1.76 | 0.833 | 1.11 | 1.04 – 1.19 | **0.002** | 1.03 | 0.95 – 1.12 | 0.427 |
| [student] * age at entry [20] | 1.81 | 1.42 – 2.30 | **<0.001** | 2.48 | 1.38 – 4.46 | **0.002** | 2.68 | 1.50 – 4.82 | **0.001** | 1.41 | 1.29 – 1.53 | **<0.001** | 1.13 | 1.01 – 1.25 | **0.025** |
| [student] * age at entry [21] | 2.09 | 1.47 – 2.97 | **<0.001** | 1.93 | 0.68 – 5.54 | 0.219 | 1.81 | 0.78 – 4.18 | 0.167 | 1.42 | 1.26 – 1.60 | **<0.001** | 1.34 | 1.16 – 1.55 | **<0.001** |
| [student] * age at entry [22] | 1.08 | 0.65 – 1.80 | 0.755 | 3.06 | 1.11 – 8.43 | **0.03** | 1.38 | 0.37 – 5.20 | 0.637 | 1.63 | 1.42 – 1.87 | **<0.001** | 1.32 | 1.12 – 1.56 | **0.001** |
| [student] * age at entry [23] | 1.77 | 1.09 – 2.87 | **0.02** | 3.31 | 0.70 – 15.70 | 0.132 | 2.22 | 0.73 – 6.82 | 0.162 | 1.8 | 1.54 – 2.11 | **<0.001** | 1.39 | 1.15 – 1.68 | **0.001** |
| [student] * age at entry [24] | 1.88 | 1.04 – 3.41 | **0.037** | 5.75 | 1.21 – 27.37 | **0.028** | 2.63 | 0.71 – 9.79 | 0.15 | 1.74 | 1.44 – 2.11 | **<0.001** | 1.63 | 1.30 – 2.03 | **<0.001** |
| [student] * study [second] | 0.95 | 0.78 – 1.15 | 0.569 | 1.02 | 0.62 – 1.69 | 0.928 | 2.01 | 1.18 – 3.41 | **0.01** | 1.16 | 1.08 – 1.24 | **<0.001** | 1.07 | 0.99 – 1.17 | 0.096 |
| [student] * study [third] | 0.79 | 0.64 – 0.99 | **0.038** | 1.04 | 0.59 – 1.83 | 0.897 | 1.17 | 0.62 – 2.20 | 0.627 | 1.1 | 1.02 – 1.18 | **0.012** | 1.1 | 1.01 – 1.20 | **0.034** |
| [student] * study [>3] | 1.29 | 0.96 – 1.74 | 0.087 | 1.42 | 0.69 – 2.92 | 0.345 | 5.36 | 2.83 – 10.13 | **<0.001** | 1.45 | 1.32 – 1.60 | **<0.001** | 1.27 | 1.14 – 1.42 | **<0.001** |
| Observations | 539905 | | | 540750 | | | 539115 | | | 521995 | | | 536635 | | |
| R^2^ Nagelkerke | 0.097 | | | 0.238 | | | 0.306 | | | 0.099 | | | 0.072 | | |

Supplementary table 4 (part 2 Poisson model for mental health diagnoses accounting for student status (No/Yes), Academic years, sex (Male/Female), deprivation, age at entry (factor), study year, comorbidities, and event before. IRR = incidence rate ratio.

|  | **EATING DISORDER** | | | **BIPOLAR DISPORDER** | | | **SCHIZOPHRENIA** | | | **ALCOHOL** | | | **DRUGS** | | |
| --- | --- | --- | --- | --- | --- | --- | --- | --- | --- | --- | --- | --- | --- | --- | --- |
| *Predictors* | *IRR* | *CI* | *p* | *IRR* | *CI* | *p* | *IRR* | *CI* | *p* | *IRR* | *CI* | *p* | *IRR* | *CI* | *p* |
| [student] | 0.67 | 0.35 – 1.28 | 0.227 | 1.38 | 0.46 – 4.15 | 0.563 | 0.02 | 0.01 – 0.08 | **<0.001** | 0.62 | 0.45 – 0.84 | **0.002** | 0.26 | 0.16 – 0.41 | **<0.001** |
| Academic year | 1 | 0.95 – 1.06 | 0.932 | 0.99 | 0.91 – 1.07 | 0.75 | 0.95 | 0.89 – 1.01 | 0.091 | 0.89 | 0.86 – 0.91 | **<0.001** | 0.93 | 0.91 – 0.96 | **<0.001** |
| Sex (female) | 3.07 | 2.44 – 3.86 | **<0.001** | 1.7 | 1.22 – 2.38 | **0.002** | 0.48 | 0.39 – 0.60 | **<0.001** | 0.72 | 0.66 – 0.78 | **<0.001** | 0.42 | 0.38 – 0.46 | **<0.001** |
| Deprivation [2] | 1.06 | 0.76 – 1.48 | 0.73 | 1.08 | 0.61 – 1.91 | 0.792 | 0.76 | 0.51 – 1.15 | 0.193 | 1.25 | 1.06 – 1.49 | **0.01** | 1.27 | 1.06 – 1.53 | **0.009** |
| Deprivation [3] | 0.85 | 0.61 – 1.19 | 0.34 | 0.89 | 0.51 – 1.54 | 0.679 | 1.08 | 0.75 – 1.54 | 0.68 | 1.24 | 1.06 – 1.47 | **0.009** | 1.27 | 1.07 – 1.52 | **0.006** |
| Deprivation [4] | 0.87 | 0.63 – 1.19 | 0.383 | 1.17 | 0.71 – 1.94 | 0.533 | 0.83 | 0.58 – 1.18 | 0.297 | 1.31 | 1.12 – 1.54 | **0.001** | 1.33 | 1.13 – 1.57 | **0.001** |
| Deprivation [5] | 0.91 | 0.68 – 1.23 | 0.544 | 1.19 | 0.73 – 1.92 | 0.484 | 1.08 | 0.77 – 1.50 | 0.666 | 1.35 | 1.16 – 1.57 | **<0.001** | 1.56 | 1.33 – 1.83 | **<0.001** |
| age at entry [19] | 0.86 | 0.69 – 1.06 | 0.148 | 1.14 | 0.82 – 1.60 | 0.439 | 0.7 | 0.56 – 0.88 | **0.003** | 0.92 | 0.84 – 1.02 | 0.107 | 0.99 | 0.90 – 1.10 | 0.856 |
| age at entry [20] | 0.71 | 0.52 – 0.97 | **0.034** | 0.77 | 0.48 – 1.24 | 0.275 | 0.6 | 0.43 – 0.84 | **0.003** | 0.75 | 0.65 – 0.86 | **<0.001** | 0.94 | 0.82 – 1.08 | 0.402 |
| age at entry [21] | 0.66 | 0.41 – 1.06 | 0.084 | 0.53 | 0.25 – 1.12 | 0.097 | 0.6 | 0.38 – 0.95 | **0.028** | 0.86 | 0.71 – 1.04 | 0.121 | 0.86 | 0.71 – 1.04 | 0.127 |
| age at entry [22] | 0.47 | 0.24 – 0.91 | **0.026** | 0.94 | 0.47 – 1.89 | 0.867 | 0.61 | 0.36 – 1.04 | 0.07 | 0.69 | 0.54 – 0.89 | **0.005** | 0.76 | 0.59 – 0.97 | **0.029** |
| age at entry [23] | 0.26 | 0.10 – 0.70 | **0.008** | 0.47 | 0.17 – 1.32 | 0.153 | 0.48 | 0.25 – 0.92 | **0.027** | 0.7 | 0.52 – 0.93 | **0.015** | 0.69 | 0.52 – 0.92 | **0.012** |
| age at entry [24] | 0.31 | 0.11 – 0.83 | **0.02** | 1.24 | 0.53 – 2.89 | 0.621 | 0.28 | 0.10 – 0.76 | **0.013** | 0.53 | 0.36 – 0.78 | **0.001** | 0.7 | 0.50 – 0.98 | **0.037** |
| Study year [second] | 1.13 | 0.90 – 1.42 | 0.283 | 1.32 | 0.90 – 1.94 | 0.155 | 1.14 | 0.88 – 1.48 | 0.324 | 0.96 | 0.86 – 1.07 | 0.45 | 1.13 | 1.01 – 1.26 | **0.035** |
| Study year [third] | 0.96 | 0.75 – 1.24 | 0.765 | 1.54 | 1.04 – 2.29 | **0.031** | 1.15 | 0.88 – 1.51 | 0.315 | 0.92 | 0.83 – 1.03 | 0.162 | 1.04 | 0.92 – 1.17 | 0.524 |
| Study year [>3] | 0.69 | 0.49 – 0.97 | **0.031** | 2.51 | 1.66 – 3.80 | **<0.001** | 1.53 | 1.13 – 2.07 | **0.005** | 0.87 | 0.75 – 1.00 | 0.05 | 1.32 | 1.16 – 1.51 | **<0.001** |
| Self-harm before | 1.72 | 1.35 – 2.19 | **<0.001** | 3.48 | 2.56 – 4.73 | **<0.001** | 1.78 | 1.27 – 2.50 | **0.001** | 2.21 | 1.95 – 2.51 | **<0.001** | 2.41 | 2.11 – 2.76 | **<0.001** |
| ASD before | 1.49 | 0.83 – 2.67 | 0.178 | 1.53 | 0.74 – 3.19 | 0.254 | 0.68 | 0.36 – 1.28 | 0.228 | 0.67 | 0.48 – 0.93 | **0.018** | 0.61 | 0.44 – 0.86 | **0.004** |
| ADHD before | 0.84 | 0.48 – 1.45 | 0.531 | 2.73 | 1.76 – 4.22 | **<0.001** | 1.61 | 1.12 – 2.33 | **0.011** | 1.55 | 1.30 – 1.83 | **<0.001** | 1.83 | 1.58 – 2.13 | **<0.001** |
| Depression before | 1.85 | 1.51 – 2.25 | **<0.001** | 5.55 | 4.01 – 7.68 | **<0.001** | 2.53 | 1.94 – 3.31 | **<0.001** | 1.59 | 1.42 – 1.77 | **<0.001** | 2.29 | 2.05 – 2.57 | **<0.001** |
| Anxiety before | 1.76 | 1.43 – 2.18 | **<0.001** | 2.22 | 1.64 – 3.00 | **<0.001** | 1.12 | 0.81 – 1.55 | 0.484 | 1.39 | 1.23 – 1.57 | **<0.001** | 1.42 | 1.25 – 1.62 | **<0.001** |
| Eating disorder before | 10.38 | 8.51 – 12.66 | **<0.001** | 1.09 | 0.62 – 1.93 | 0.757 | 1.47 | 0.82 – 2.65 | 0.198 | 1.46 | 1.16 – 1.84 | **0.001** | 0.86 | 0.63 – 1.19 | 0.369 |
| Bipolar before | 0.88 | 0.21 – 3.65 | 0.855 | 36.11 | 22.07 – 59.06 | **<0.001** | 0.94 | 0.43 – 2.06 | 0.874 | 1.37 | 0.72 – 2.60 | 0.341 | 1.17 | 0.62 – 2.21 | 0.628 |
| Schizophrenia before | 0.49 | 0.07 – 3.70 | 0.491 | 1.34 | 0.55 – 3.30 | 0.521 | 45.04 | 29.55 – 68.63 | **<0.001** | 1.38 | 0.91 – 2.08 | 0.125 | 2.25 | 1.67 – 3.04 | **<0.001** |
| Alcohol before | 1 | 0.69 – 1.45 | 0.989 | 1.5 | 1.01 – 2.23 | **0.046** | 0.88 | 0.59 – 1.30 | 0.517 | 2.73 | 2.38 – 3.13 | **<0.001** | 1.65 | 1.42 – 1.92 | **<0.001** |
| Drugs before | 1.31 | 0.79 – 2.17 | 0.299 | 0.88 | 0.50 – 1.55 | 0.66 | 4.02 | 2.66 – 6.06 | **<0.001** | 2.1 | 1.76 – 2.52 | **<0.001** | 4.94 | 4.21 – 5.79 | **<0.001** |
| [student] * academic year | 0.98 | 0.90 – 1.07 | 0.684 | 0.94 | 0.80 – 1.10 | 0.435 | 1.25 | 1.04 – 1.50 | **0.017** | 1.15 | 1.09 – 1.21 | **<0.001** | 1 | 0.92 – 1.09 | 0.94 |
| [student] * sex (female) | 1.88 | 1.20 – 2.95 | **0.006** | 0.97 | 0.52 – 1.82 | 0.919 | 1.34 | 0.71 – 2.54 | 0.369 | 1.01 | 0.85 – 1.19 | 0.944 | 0.94 | 0.70 – 1.27 | 0.701 |
| [student] * Deprivation [2] | 0.84 | 0.53 – 1.34 | 0.46 | 0.63 | 0.25 – 1.58 | 0.325 | 0.95 | 0.31 – 2.93 | 0.927 | 1 | 0.76 – 1.32 | 0.979 | 1.42 | 0.92 – 2.21 | 0.117 |
| [student] * Deprivation [3] | 0.76 | 0.46 – 1.24 | 0.269 | 1.06 | 0.45 – 2.52 | 0.891 | 0.91 | 0.32 – 2.56 | 0.854 | 0.87 | 0.66 – 1.14 | 0.304 | 1.08 | 0.68 – 1.70 | 0.756 |
| [student] * Deprivation [4] | 0.92 | 0.57 – 1.48 | 0.731 | 0.47 | 0.18 – 1.25 | 0.129 | 1.9 | 0.74 – 4.89 | 0.184 | 0.91 | 0.69 – 1.20 | 0.502 | 1.29 | 0.82 – 2.02 | 0.27 |
| [student] * Deprivation [5] | 0.71 | 0.43 – 1.17 | 0.174 | 0.74 | 0.32 – 1.72 | 0.489 | 1.51 | 0.58 – 3.94 | 0.401 | 0.99 | 0.76 – 1.31 | 0.963 | 0.85 | 0.52 – 1.39 | 0.521 |
| [student] * age at entry [19] | 1.14 | 0.80 – 1.61 | 0.467 | 0.69 | 0.33 – 1.47 | 0.341 | 2.18 | 0.90 – 5.28 | 0.084 | 1.09 | 0.90 – 1.32 | 0.403 | 0.97 | 0.67 – 1.41 | 0.878 |
| [student] * age at entry [20] | 1.51 | 0.94 – 2.43 | 0.09 | 2.08 | 0.91 – 4.76 | 0.084 | 2.53 | 0.81 – 7.86 | 0.11 | 1.21 | 0.92 – 1.59 | 0.175 | 1.81 | 1.19 – 2.75 | **0.005** |
| [student] * age at entry [21] | 1.43 | 0.69 – 2.96 | 0.34 | 1.19 | 0.31 – 4.60 | 0.803 | 5.02 | 1.50 – 16.81 | **0.009** | 0.95 | 0.64 – 1.41 | 0.805 | 2.26 | 1.34 – 3.82 | **0.002** |
| [student] * age at entry [22] | 1.77 | 0.70 – 4.48 | 0.229 | 1.95 | 0.63 – 6.08 | 0.247 | 7.98 | 2.55 – 24.97 | **<0.001** | 1 | 0.60 – 1.65 | 0.991 | 1.97 | 1.03 – 3.77 | **0.04** |
| [student] * age at entry [23] | 1.73 | 0.45 – 6.61 | 0.425 | 1.3 | 0.25 – 6.74 | 0.755 | 5.32 | 1.16 – 24.31 | **0.031** | 1.24 | 0.73 – 2.09 | 0.428 | 1.8 | 0.84 – 3.84 | 0.13 |
| [student] * age at entry [24] | 1.86 | 0.48 – 7.12 | 0.366 | 1.94 | 0.57 – 6.59 | 0.289 | 4.79 | 0.46 – 49.61 | 0.189 | 1.26 | 0.64 – 2.48 | 0.506 | 2.14 | 0.92 – 5.00 | 0.079 |
| [student] * study [second] | 0.96 | 0.66 – 1.39 | 0.829 | 1.16 | 0.54 – 2.48 | 0.698 | 2.82 | 1.06 – 7.51 | **0.038** | 0.8 | 0.66 – 0.99 | **0.036** | 0.71 | 0.49 – 1.01 | 0.058 |
| [student] * study [third] | 1.23 | 0.83 – 1.82 | 0.302 | 1.26 | 0.58 – 2.71 | 0.561 | 2.41 | 0.84 – 6.87 | 0.101 | 0.69 | 0.55 – 0.87 | **0.001** | 0.84 | 0.58 – 1.22 | 0.35 |
| [student] * study [>3] | 1.66 | 0.94 – 2.95 | 0.082 | 1.62 | 0.70 – 3.76 | 0.262 | 4.57 | 1.60 – 13.09 | **0.005** | 0.98 | 0.73 – 1.33 | 0.907 | 1.06 | 0.68 – 1.66 | 0.793 |
| Observations | 540705 | | | 540795 | | | 540755 | | | 540530 | | | 540440 | | |
| R^2^ Nagelkerke | 0.087 | | | 0.192 | | | 0.173 | | | 0.041 | | | 0.12 | | |
