# Supplementary material for: Trends in incidence of self-harm, neurodevelopmental and mental health conditions among university students compared with the general population: nationwide electronic data linkage study in Wales
Source: Br J Psychiatry. 2024 Sep;225(3):389–400. doi: 10.1192/bjp.2024.90 (PMC11536190; doi:10.1192/bjp.2024.90)
Supplement: John et al. supplementary material 7 — John et al. supplementary material [file S0007125024000904sup007.docx]

Supplementary table 5 (part 1) Poisson model for mental health diagnoses accounting for student status (No/Yes), Academic years (factor), sex (Male/Female), deprivation, age at entry, study year, comorbidities, and event before. IRR = incidence rate ratio.

|  | **SELF-HARM** | | | **ASD** | | | **ADHD** | | | **DEPRESSION** | | | **ANXIETY** | | |
| --- | --- | --- | --- | --- | --- | --- | --- | --- | --- | --- | --- | --- | --- | --- | --- |
| *Predictors* | *IRR* | *CI* | *p* | *IRR* | *CI* | *p* | *IRR* | *CI* | *p* | *IRR* | *CI* | *p* | *IRR* | *CI* | *p* |
| [student] | 0.03 | 0.01 – 0.08 | **<0.001** | 0 | 0.00 – 0.02 | **<0.001** | 0.01 | 0.00 – 0.12 | **<0.001** | 0.09 | 0.07 – 0.13 | **<0.001** | 0.23 | 0.15 – 0.35 | **<0.001** |
| Academic year [2] | 0.89 | 0.80 – 0.99 | **0.037** | 1.44 | 1.05 – 1.97 | **0.025** | 0.5 | 0.39 – 0.65 | **<0.001** | 0.75 | 0.71 – 0.79 | **<0.001** | 1.06 | 0.99 – 1.14 | 0.099 |
| Academic year [3] | 0.84 | 0.75 – 0.93 | **0.001** | 1.18 | 0.86 – 1.63 | 0.308 | 0.58 | 0.46 – 0.74 | **<0.001** | 0.86 | 0.82 – 0.91 | **<0.001** | 1.13 | 1.05 – 1.21 | **0.001** |
| Academic year [4] | 0.91 | 0.82 – 1.01 | 0.088 | 1.36 | 1.00 – 1.85 | 0.053 | 0.61 | 0.48 – 0.77 | **<0.001** | 0.95 | 0.90 – 0.99 | **0.023** | 1.37 | 1.28 – 1.47 | **<0.001** |
| Academic year [5] | 0.85 | 0.76 – 0.94 | **0.003** | 1.63 | 1.21 – 2.20 | **0.001** | 0.65 | 0.52 – 0.81 | **<0.001** | 0.93 | 0.89 – 0.97 | **0.002** | 1.5 | 1.40 – 1.60 | **<0.001** |
| Academic year [6] | 0.89 | 0.80 – 0.99 | **0.036** | 1.38 | 1.01 – 1.87 | **0.041** | 0.65 | 0.52 – 0.82 | **<0.001** | 0.97 | 0.93 – 1.02 | 0.265 | 1.62 | 1.51 – 1.73 | **<0.001** |
| Sex (female) | 0.94 | 0.88 – 1.01 | 0.077 | 0.87 | 0.72 – 1.05 | 0.146 | 0.84 | 0.70 – 1.01 | 0.067 | 1.85 | 1.79 – 1.90 | **<0.001** | 1.74 | 1.68 – 1.81 | **<0.001** |
| Deprivation [2] | 1.19 | 1.04 – 1.36 | **0.011** | 1.28 | 0.95 – 1.72 | 0.105 | 1.26 | 0.93 – 1.70 | 0.137 | 1.05 | 1.00 – 1.11 | 0.074 | 1 | 0.93 – 1.07 | 0.995 |
| Deprivation [3] | 1.29 | 1.14 – 1.46 | **<0.001** | 1.38 | 1.05 – 1.82 | **0.021** | 1.16 | 0.87 – 1.54 | 0.318 | 1.12 | 1.06 – 1.18 | **<0.001** | 1.05 | 0.98 – 1.12 | 0.181 |
| Deprivation [4] | 1.31 | 1.17 – 1.48 | **<0.001** | 1.17 | 0.89 – 1.53 | 0.262 | 1.19 | 0.91 – 1.57 | 0.198 | 1.23 | 1.17 – 1.29 | **<0.001** | 1.11 | 1.04 – 1.18 | **0.001** |
| Deprivation [5] | 1.54 | 1.38 – 1.72 | **<0.001** | 1.27 | 0.97 – 1.65 | 0.08 | 1.46 | 1.13 – 1.89 | **0.004** | 1.39 | 1.33 – 1.46 | **<0.001** | 1.1 | 1.04 – 1.17 | **0.002** |
| Age at entry | 0.86 | 0.83 – 0.88 | **<0.001** | 0.82 | 0.76 – 0.88 | **<0.001** | 0.86 | 0.80 – 0.92 | **<0.001** | 0.95 | 0.94 – 0.96 | **<0.001** | 0.97 | 0.95 – 0.98 | **<0.001** |
| Study year [second] | 0.96 | 0.89 – 1.04 | 0.35 | 1.01 | 0.84 – 1.22 | 0.913 | 1.19 | 1.00 – 1.42 | **0.05** | 1.14 | 1.10 – 1.18 | **<0.001** | 1.1 | 1.04 – 1.15 | **<0.001** |
| Study year [third] | 0.89 | 0.82 – 0.96 | **0.005** | 0.82 | 0.66 – 1.01 | 0.065 | 1.11 | 0.92 – 1.34 | 0.258 | 1.21 | 1.17 – 1.26 | **<0.001** | 1.19 | 1.13 – 1.25 | **<0.001** |
| Study year [>3] | 0.75 | 0.67 – 0.83 | **<0.001** | 0.87 | 0.66 – 1.13 | 0.282 | 0.79 | 0.61 – 1.02 | 0.074 | 1.25 | 1.19 – 1.30 | **<0.001** | 1.21 | 1.15 – 1.29 | **<0.001** |
| Self-harm before | 3.53 | 3.24 – 3.84 | **<0.001** | 1.61 | 1.26 – 2.07 | **<0.001** | 1.46 | 1.18 – 1.80 | **<0.001** | 1.67 | 1.60 – 1.74 | **<0.001** | 1.32 | 1.25 – 1.40 | **<0.001** |
| ASD before | 0.97 | 0.78 – 1.20 | 0.771 | 55.48 | 45.81 – 67.19 | **<0.001** | 1.2 | 0.94 – 1.52 | 0.139 | 0.84 | 0.75 – 0.94 | **0.002** | 1.23 | 1.09 – 1.38 | **0.001** |
| ADHD before | 1.76 | 1.56 – 1.98 | **<0.001** | 1.69 | 1.36 – 2.09 | **<0.001** | 95.51 | 79.56 – 114.65 | **<0.001** | 1.46 | 1.36 – 1.56 | **<0.001** | 1.23 | 1.12 – 1.35 | **<0.001** |
| Depression before | 2.64 | 2.44 – 2.86 | **<0.001** | 1.7 | 1.39 – 2.08 | **<0.001** | 1.56 | 1.30 – 1.89 | **<0.001** | 3.76 | 3.64 – 3.88 | **<0.001** | 1.99 | 1.90 – 2.07 | **<0.001** |
| Anxiety before | 1.39 | 1.27 – 1.51 | **<0.001** | 1.46 | 1.19 – 1.81 | **<0.001** | 1.27 | 1.03 – 1.56 | **0.026** | 1.58 | 1.52 – 1.64 | **<0.001** | 3.41 | 3.27 – 3.56 | **<0.001** |
| Eating disorder before | 1.48 | 1.26 – 1.73 | **<0.001** | 1.78 | 1.23 – 2.57 | **0.002** | 0.9 | 0.56 – 1.44 | 0.656 | 1.2 | 1.11 – 1.30 | **<0.001** | 1.24 | 1.13 – 1.37 | **<0.001** |
| Bipolar before | 1.24 | 0.81 – 1.91 | 0.325 | 1.59 | 0.68 – 3.71 | 0.287 | 0.94 | 0.29 – 3.02 | 0.912 | 0.68 | 0.50 – 0.93 | **0.014** | 1.16 | 0.85 – 1.58 | 0.353 |
| Schizophrenia before | 1.76 | 1.34 – 2.32 | **<0.001** | 1.83 | 0.91 – 3.69 | 0.09 | 0.97 | 0.52 – 1.84 | 0.937 | 1.21 | 0.99 – 1.47 | 0.06 | 1.55 | 1.24 – 1.93 | **<0.001** |
| Alcohol before | 1.79 | 1.61 – 1.99 | **<0.001** | 0.63 | 0.39 – 1.03 | 0.067 | 1.04 | 0.80 – 1.36 | 0.751 | 1.2 | 1.13 – 1.27 | **<0.001** | 1.15 | 1.07 – 1.25 | **<0.001** |
| Drugs before | 1.65 | 1.45 – 1.87 | **<0.001** | 0.67 | 0.34 – 1.31 | 0.242 | 1.54 | 1.17 – 2.03 | **0.002** | 1.43 | 1.32 – 1.54 | **<0.001** | 1.5 | 1.36 – 1.65 | **<0.001** |
| [student] * academic year [2] | 1.05 | 0.78 – 1.40 | 0.765 | 0.76 | 0.24 – 2.38 | 0.64 | 0.72 | 0.29 – 1.78 | 0.481 | 0.98 | 0.89 – 1.08 | 0.644 | 0.99 | 0.88 – 1.13 | 0.931 |
| [student] * academic year [3] | 1.33 | 1.00 – 1.76 | 0.05 | 1.89 | 0.69 – 5.15 | 0.214 | 1.15 | 0.55 – 2.42 | 0.704 | 0.95 | 0.86 – 1.04 | 0.279 | 1.05 | 0.93 – 1.19 | 0.414 |
| [student] * academic year [4] | 1.14 | 0.86 – 1.52 | 0.356 | 2.91 | 1.14 – 7.42 | **0.025** | 1.72 | 0.89 – 3.33 | 0.11 | 0.97 | 0.88 – 1.06 | 0.463 | 1 | 0.89 – 1.13 | 0.999 |
| [student] * academic year [5] | 1.42 | 1.08 – 1.87 | **0.013** | 1.75 | 0.67 – 4.56 | 0.255 | 1.38 | 0.70 – 2.72 | 0.351 | 1.03 | 0.94 – 1.12 | 0.586 | 0.96 | 0.86 – 1.08 | 0.539 |
| [student] * academic year [6] | 1.44 | 1.10 – 1.90 | **0.009** | 2.81 | 1.10 – 7.17 | **0.031** | 2.47 | 1.34 – 4.54 | **0.004** | 1.13 | 1.03 – 1.23 | **0.01** | 1.02 | 0.91 – 1.15 | 0.676 |
| [student] * sex (female) | 1.74 | 1.46 – 2.08 | **<0.001** | 0.54 | 0.35 – 0.86 | **0.008** | 0.87 | 0.57 – 1.31 | 0.498 | 0.82 | 0.78 – 0.87 | **<0.001** | 1 | 0.93 – 1.07 | 0.972 |
| [student] * Deprivation [2] | 0.9 | 0.70 – 1.16 | 0.423 | 1.12 | 0.55 – 2.26 | 0.762 | 0.85 | 0.45 – 1.59 | 0.611 | 0.91 | 0.83 – 0.99 | **0.031** | 0.99 | 0.89 – 1.10 | 0.88 |
| [student] * Deprivation [3] | 0.79 | 0.61 – 1.02 | 0.066 | 1.24 | 0.63 – 2.43 | 0.53 | 0.83 | 0.44 – 1.57 | 0.568 | 0.89 | 0.82 – 0.98 | **0.012** | 0.92 | 0.83 – 1.02 | 0.133 |
| [student] * Deprivation [4] | 0.77 | 0.59 – 1.00 | **0.048** | 2.09 | 1.10 – 3.99 | **0.025** | 0.92 | 0.48 – 1.74 | 0.79 | 0.9 | 0.82 – 0.98 | **0.015** | 0.88 | 0.79 – 0.98 | **0.018** |
| [student] * Deprivation [5] | 0.84 | 0.65 – 1.08 | 0.181 | 1.41 | 0.69 – 2.86 | 0.346 | 0.67 | 0.35 – 1.30 | 0.237 | 0.79 | 0.72 – 0.86 | **<0.001** | 0.95 | 0.85 – 1.05 | 0.308 |
| [student] * age at entry | 1.14 | 1.08 – 1.20 | **<0.001** | 1.33 | 1.16 – 1.53 | **<0.001** | 1.23 | 1.07 – 1.41 | **0.003** | 1.12 | 1.10 – 1.14 | **<0.001** | 1.08 | 1.05 – 1.10 | **<0.001** |
| [student] * study [second] | 0.94 | 0.78 – 1.15 | 0.563 | 1.04 | 0.63 – 1.71 | 0.873 | 1.97 | 1.15 – 3.36 | **0.013** | 1.16 | 1.08 – 1.24 | **<0.001** | 1.07 | 0.99 – 1.17 | 0.096 |
| [student] * study [third] | 0.79 | 0.63 – 0.98 | **0.036** | 1.04 | 0.59 – 1.84 | 0.882 | 1.18 | 0.63 – 2.22 | 0.613 | 1.1 | 1.02 – 1.18 | **0.012** | 1.1 | 1.01 – 1.20 | **0.035** |
| [student] * study [>3] | 1.3 | 0.97 – 1.74 | 0.083 | 1.43 | 0.69 – 2.94 | 0.333 | 5.28 | 2.77 – 10.05 | **<0.001** | 1.46 | 1.33 – 1.60 | **<0.001** | 1.27 | 1.14 – 1.43 | **<0.001** |
| Observations | 539905 | | | 540750 | | | 539115 | | | 521995 | | | 536635 | | |
| R^2^ Nagelkerke | 0.097 | | | 0.239 | | | 0.308 | | | 0.1 | | | 0.072 | | |

Supplementary table 5 (part 2) Poisson model for mental health diagnoses accounting for student status (No/Yes), Academic years (factor), sex (Male/Female), deprivation, age at entry, study year, comorbidities, and event before. IRR = incidence rate ratio.

|  | **EATING DISORDER** | | | **BIPOLAR DISPORDER** | | | **SCHIZOPHRENIA** | | | **ALCOHOL** | | | **DRUGS** | | |
| --- | --- | --- | --- | --- | --- | --- | --- | --- | --- | --- | --- | --- | --- | --- | --- |
| *Predictors* | *IRR* | *CI* | *p* | *IRR* | *CI* | *p* | *IRR* | *CI* | *p* | *IRR* | *CI* | *p* | *IRR* | *CI* | *p* |
| [student] | 0.1 | 0.01 – 0.90 | **0.04** | 0.05 | 0.00 – 1.48 | 0.083 | 0 | 0.00 – 0.00 | **<0.001** | 0.39 | 0.12 – 1.31 | 0.13 | 0.01 | 0.00 – 0.06 | **<0.001** |
| Academic year [2] | 0.8 | 0.56 – 1.14 | 0.217 | 1.49 | 0.85 – 2.61 | 0.161 | 0.71 | 0.50 – 1.00 | 0.051 | 0.85 | 0.74 – 0.97 | **0.018** | 0.74 | 0.64 – 0.86 | **<0.001** |
| Academic year [3] | 1.26 | 0.91 – 1.73 | 0.16 | 1.51 | 0.87 – 2.62 | 0.143 | 0.77 | 0.55 – 1.07 | 0.124 | 0.73 | 0.63 – 0.84 | **<0.001** | 0.8 | 0.70 – 0.93 | **0.003** |
| Academic year [4] | 1.08 | 0.78 – 1.50 | 0.638 | 1.56 | 0.91 – 2.66 | 0.103 | 0.74 | 0.53 – 1.04 | 0.08 | 0.69 | 0.60 – 0.79 | **<0.001** | 0.7 | 0.61 – 0.81 | **<0.001** |
| Academic year [5] | 0.99 | 0.72 – 1.38 | 0.969 | 1.06 | 0.60 – 1.86 | 0.843 | 0.78 | 0.57 – 1.09 | 0.144 | 0.64 | 0.56 – 0.74 | **<0.001** | 0.74 | 0.64 – 0.85 | **<0.001** |
| Academic year [6] | 0.95 | 0.68 – 1.32 | 0.755 | 1.23 | 0.72 – 2.12 | 0.451 | 0.66 | 0.47 – 0.94 | **0.02** | 0.51 | 0.44 – 0.59 | **<0.001** | 0.64 | 0.56 – 0.75 | **<0.001** |
| Sex (female) | 3.07 | 2.44 – 3.86 | **<0.001** | 1.69 | 1.21 – 2.35 | **0.002** | 0.48 | 0.38 – 0.60 | **<0.001** | 0.72 | 0.65 – 0.78 | **<0.001** | 0.42 | 0.38 – 0.46 | **<0.001** |
| Deprivation [2] | 1.06 | 0.76 – 1.48 | 0.734 | 1.11 | 0.63 – 1.96 | 0.723 | 0.77 | 0.51 – 1.15 | 0.203 | 1.26 | 1.06 – 1.49 | **0.01** | 1.27 | 1.06 – 1.53 | **0.01** |
| Deprivation [3] | 0.85 | 0.61 – 1.18 | 0.335 | 0.88 | 0.51 – 1.52 | 0.642 | 1.09 | 0.76 – 1.55 | 0.636 | 1.24 | 1.06 – 1.47 | **0.009** | 1.28 | 1.07 – 1.52 | **0.006** |
| Deprivation [4] | 0.87 | 0.63 – 1.19 | 0.379 | 1.2 | 0.73 – 1.98 | 0.476 | 0.84 | 0.59 – 1.19 | 0.317 | 1.31 | 1.12 – 1.54 | **0.001** | 1.33 | 1.13 – 1.57 | **0.001** |
| Deprivation [5] | 0.91 | 0.68 – 1.23 | 0.537 | 1.22 | 0.75 – 1.96 | 0.425 | 1.08 | 0.78 – 1.50 | 0.645 | 1.35 | 1.16 – 1.57 | **<0.001** | 1.56 | 1.33 – 1.83 | **<0.001** |
| Age at entry | 0.83 | 0.76 – 0.89 | **<0.001** | 0.93 | 0.83 – 1.03 | 0.159 | 0.85 | 0.78 – 0.92 | **<0.001** | 0.91 | 0.88 – 0.95 | **<0.001** | 0.94 | 0.91 – 0.97 | **<0.001** |
| Study year [second] | 1.14 | 0.90 – 1.43 | 0.276 | 1.31 | 0.89 – 1.92 | 0.177 | 1.15 | 0.88 – 1.49 | 0.306 | 0.96 | 0.86 – 1.07 | 0.451 | 1.13 | 1.01 – 1.26 | **0.032** |
| Study year [third] | 0.96 | 0.75 – 1.24 | 0.777 | 1.54 | 1.04 – 2.28 | **0.03** | 1.15 | 0.87 – 1.51 | 0.323 | 0.92 | 0.83 – 1.03 | 0.168 | 1.04 | 0.92 – 1.17 | 0.522 |
| Study year [>3] | 0.69 | 0.49 – 0.97 | **0.033** | 2.46 | 1.63 – 3.72 | **<0.001** | 1.53 | 1.13 – 2.06 | **0.006** | 0.87 | 0.75 – 1.00 | 0.052 | 1.32 | 1.16 – 1.51 | **<0.001** |
| Self-harm before | 1.72 | 1.35 – 2.19 | **<0.001** | 3.42 | 2.50 – 4.67 | **<0.001** | 1.77 | 1.26 – 2.48 | **0.001** | 2.21 | 1.95 – 2.51 | **<0.001** | 2.41 | 2.11 – 2.76 | **<0.001** |
| ASD before | 1.52 | 0.85 – 2.72 | 0.161 | 1.43 | 0.68 – 3.00 | 0.339 | 0.69 | 0.37 – 1.28 | 0.24 | 0.67 | 0.48 – 0.93 | **0.018** | 0.61 | 0.44 – 0.86 | **0.004** |
| ADHD before | 0.84 | 0.48 – 1.46 | 0.535 | 2.63 | 1.71 – 4.05 | **<0.001** | 1.6 | 1.11 – 2.32 | **0.012** | 1.55 | 1.30 – 1.83 | **<0.001** | 1.84 | 1.58 – 2.13 | **<0.001** |
| Depression before | 1.85 | 1.52 – 2.26 | **<0.001** | 5.56 | 4.01 – 7.70 | **<0.001** | 2.53 | 1.94 – 3.30 | **<0.001** | 1.59 | 1.42 – 1.77 | **<0.001** | 2.3 | 2.05 – 2.57 | **<0.001** |
| Anxiety before | 1.76 | 1.42 – 2.17 | **<0.001** | 2.22 | 1.65 – 3.00 | **<0.001** | 1.13 | 0.82 – 1.55 | 0.454 | 1.39 | 1.22 – 1.57 | **<0.001** | 1.42 | 1.25 – 1.61 | **<0.001** |
| Eating disorder before | 10.32 | 8.46 – 12.58 | **<0.001** | 1.11 | 0.63 – 1.94 | 0.714 | 1.49 | 0.83 – 2.68 | 0.186 | 1.45 | 1.15 – 1.83 | **0.002** | 0.87 | 0.63 – 1.19 | 0.384 |
| Bipolar before | 0.85 | 0.20 – 3.53 | 0.821 | 34.21 | 20.95 – 55.86 | **<0.001** | 0.98 | 0.44 – 2.19 | 0.954 | 1.39 | 0.73 – 2.65 | 0.31 | 1.16 | 0.61 – 2.20 | 0.649 |
| Schizophrenia before | 0.48 | 0.06 – 3.60 | 0.476 | 1.45 | 0.60 – 3.49 | 0.412 | 44.29 | 29.18 – 67.22 | **<0.001** | 1.37 | 0.91 – 2.07 | 0.13 | 2.24 | 1.66 – 3.02 | **<0.001** |
| Alcohol before | 1 | 0.69 – 1.46 | 0.989 | 1.48 | 0.99 – 2.22 | 0.055 | 0.88 | 0.59 – 1.30 | 0.512 | 2.73 | 2.38 – 3.13 | **<0.001** | 1.65 | 1.42 – 1.92 | **<0.001** |
| Drugs before | 1.3 | 0.78 – 2.15 | 0.313 | 0.87 | 0.50 – 1.54 | 0.641 | 3.99 | 2.65 – 6.00 | **<0.001** | 2.1 | 1.76 – 2.51 | **<0.001** | 4.94 | 4.21 – 5.79 | **<0.001** |
| [student] * academic year [2] | 1.13 | 0.66 – 1.91 | 0.659 | 0.96 | 0.37 – 2.48 | 0.937 | 3.49 | 1.06 – 11.54 | **0.04** | 1.35 | 1.01 – 1.80 | **0.041** | 0.99 | 0.62 – 1.57 | 0.959 |
| [student] * academic year [3] | 0.61 | 0.36 – 1.02 | 0.059 | 0.89 | 0.35 – 2.29 | 0.81 | 1.04 | 0.23 – 4.77 | 0.955 | 1.53 | 1.14 – 2.04 | **0.005** | 1.16 | 0.74 – 1.80 | 0.522 |
| [student] * academic year [4] | 0.57 | 0.33 – 0.98 | **0.042** | 0.32 | 0.10 – 0.99 | **0.047** | 3.65 | 1.13 – 11.75 | **0.03** | 1.83 | 1.37 – 2.43 | **<0.001** | 0.86 | 0.53 – 1.41 | 0.551 |
| [student] * academic year [5] | 0.83 | 0.50 – 1.39 | 0.485 | 0.75 | 0.27 – 2.11 | 0.584 | 2.97 | 0.90 – 9.82 | 0.075 | 1.98 | 1.49 – 2.64 | **<0.001** | 0.99 | 0.62 – 1.58 | 0.967 |
| [student] * academic year [6] | 1.03 | 0.62 – 1.71 | 0.904 | 0.81 | 0.30 – 2.17 | 0.672 | 4.1 | 1.27 – 13.26 | **0.018** | 2.01 | 1.48 – 2.74 | **<0.001** | 1.08 | 0.67 – 1.76 | 0.745 |
| [student] * sex (female) | 1.87 | 1.19 – 2.94 | **0.007** | 0.99 | 0.53 – 1.87 | 0.986 | 1.31 | 0.69 – 2.47 | 0.414 | 1.01 | 0.85 – 1.19 | 0.94 | 0.93 | 0.69 – 1.25 | 0.64 |
| [student] * Deprivation [2] | 0.84 | 0.53 – 1.34 | 0.469 | 0.66 | 0.26 – 1.65 | 0.37 | 0.98 | 0.31 – 3.06 | 0.97 | 1 | 0.76 – 1.32 | 0.984 | 1.42 | 0.92 – 2.21 | 0.116 |
| [student] * Deprivation [3] | 0.76 | 0.46 – 1.24 | 0.264 | 1.12 | 0.47 – 2.65 | 0.796 | 0.92 | 0.33 – 2.59 | 0.88 | 0.87 | 0.66 – 1.14 | 0.304 | 1.08 | 0.68 – 1.71 | 0.738 |
| [student] * Deprivation [4] | 0.93 | 0.58 – 1.49 | 0.765 | 0.49 | 0.19 – 1.27 | 0.142 | 2.02 | 0.79 – 5.16 | 0.144 | 0.91 | 0.69 – 1.19 | 0.494 | 1.3 | 0.82 – 2.04 | 0.26 |
| [student] * Deprivation [5] | 0.71 | 0.43 – 1.17 | 0.184 | 0.76 | 0.32 – 1.78 | 0.523 | 1.61 | 0.62 – 4.21 | 0.329 | 0.99 | 0.75 – 1.30 | 0.953 | 0.87 | 0.53 – 1.42 | 0.577 |
| [student] * age at entry | 1.12 | 1.00 – 1.26 | **0.041** | 1.19 | 1.01 – 1.41 | **0.041** | 1.43 | 1.20 – 1.70 | **<0.001** | 1.03 | 0.97 – 1.09 | 0.374 | 1.18 | 1.09 – 1.28 | **<0.001** |
| [student] * study [second] | 0.95 | 0.65 – 1.38 | 0.796 | 1.15 | 0.54 – 2.47 | 0.711 | 2.95 | 1.12 – 7.79 | **0.029** | 0.81 | 0.66 – 0.99 | **0.038** | 0.7 | 0.49 – 1.00 | 0.053 |
| [student] * study [third] | 1.22 | 0.82 – 1.80 | 0.33 | 1.24 | 0.58 – 2.66 | 0.581 | 2.41 | 0.84 – 6.96 | 0.103 | 0.69 | 0.55 – 0.87 | **0.002** | 0.83 | 0.57 – 1.21 | 0.343 |
| [student] * study [>3] | 1.65 | 0.93 – 2.92 | 0.088 | 1.68 | 0.73 – 3.86 | 0.225 | 5.04 | 1.79 – 14.22 | **0.002** | 0.98 | 0.73 – 1.32 | 0.896 | 1.08 | 0.69 – 1.68 | 0.745 |
| Observations | 540705 | | | 540795 | | | 540755 | | | 540530 | | | 540440 | | |
| R^2^ Nagelkerke | 0.088 | | | 0.19 | | | 0.173 | | | 0.041 | | | 0.12 | | |
