# Supplementary material for: Trends in incidence of self-harm, neurodevelopmental and mental health conditions among university students compared with the general population: nationwide electronic data linkage study in Wales
Source: Br J Psychiatry. 2024 Sep;225(3):389–400. doi: 10.1192/bjp.2024.90 (PMC11536190; doi:10.1192/bjp.2024.90)
Supplement: John et al. supplementary material 8 — John et al. supplementary material [file S0007125024000904sup008.docx]

Supplementary table 6 – Crude incidence rate (95% CI) for self-harm, ND, and mental health conditions for students and non-students

|  | ***Students*** | | | ***Non-students*** | | |
| --- | --- | --- | --- | --- | --- | --- |
| ***Events*** | ***Count*** | ***PYAR*** | ***Incidence per 1000 PYAR (95% CI)*** | ***Count*** | ***PYAR*** | ***Incidence per 1000 PYAR (95% CI)*** |
| ***Self-harm*** | 710 | 168,635 | 4·19 (3·88 – 4·51) | 4,190 | 327,420 | 12·55 (12·17 – 12·93) |
| ***ASD*** | 110 | 169,180 | 0·64 (0·53 – 0·78) | 695 | 331,125 | 2·04 (1·89 – 2·20) |
| ***ADHD*** | 180 | 169,040 | 1·06 (0·91 – 1·23) | 1,370 | 329,470 | 3·97 (3·76 – 4·19) |
| ***Depression*** | 7,870 | 160,580 | 48·96 (47·88 – 50·05) | 24,115 | 301,920 | 79·41 (78·41 – 80·41) |
| ***Anxiety*** | 5,490 | 164,260 | 42·46 (41·46 – 43·46) | 12,185 | 319,845 | 37·75 (37·09 – 38·42) |
| ***Eating disorder*** | 285 | 169,005 | 1·70 (1·51 – 1·91) | 465 | 331,325 | 1·46 (1·34 – 1·60) |
| ***Bipolar disorder*** | 75 | 169,215 | 0·44 (0·35 – 0·55) | 220 | 331,620 | 0·72 (0·63 – 0·81) |
| ***Schizophrenia spectrum disorder*** | 45 | 169,245 | 0·27 (0·20 – 0·36) | 435 | 331,390 | 1·37 (1·24 – 1·50) |
| ***Alcohol misuse*** | 745 | 168,605 | 4·39 (4·08 – 4·72) | 2,200 | 329,585 | 6·63 (6·36 – 6·91) |
| ***Drugs misuse*** | 215 | 169,105 | 1·26 (1·09 – 1·44) | 2,175 | 329,595 | 6·57 (6·30 – 6·85) |
